# Abnormal redox balance at membrane contact sites causes axonopathy in *GDAP1*-related Charcot-Marie-Tooth disease

**DOI:** 10.21203/rs.3.rs-5682984/v1

**Published:** 2024-12-31

**Authors:** Lara Cantarero, Mònica Roldán, María Rodríguez-Sanz, Angela J. Mathison, Yaiza Díaz-Osorio, Jordi Pijuan, Marcos Frías, Raul Urrutia, Janet Hoenicka, Francesc Palau

**Affiliations:** 1 Laboratory of Neurogenetics and Molecular Medicine, Center for Genomic Sciences in Medicine, Institut de Recerca Sant Joan de Déu, Barcelona, Spain; 2 Centro de Investigación Biomédica en Red de Enfermedades Raras (CIBERER), Barcelona, Spain; 3 Confocal Microscopy Unit, Hospital Sant Joan de Déu, Barcelona, Spain; 4 Institut de Recerca Sant Joan de Déu (IRSJD), Barcelona, Spain; 5 Linda T. and John A. Mellowes Center for Genomic Sciences and Precision Medicine, Medical College of Wisconsin, 8701 Watertown Plank Road, Milwaukee, WI, 53226, USA; 6 Department of Surgery, Medical College of Wisconsin, 8701 Watertown Plank Road, Milwaukee, WI, 53226, USA; 7 Únicas SJD Center, Hospital Sant Joan de Déu, Barcelona, Spain; 8 Division of Pediatrics, Faculty of Medicine and Health Sciences, University of Barcelona, Barcelona, Spain

## Abstract

Pathogenic variants of *GDAP1* cause Charcot-Marie-Tooth disease (CMT), an inherited neuropathy characterized by axonal degeneration. GDAP1, an atypical glutathione S-transferase, localizes to the outer mitochondrial membrane (OMM), regulating this organelle's dynamics, transport, and membrane contact sites (MCSs). It has been proposed that GDAP1 functions as a cellular redox sensor. However, its precise contribution to redox homeostasis remains poorly understood, as does the possible redox regulation at mitochondrial MCSs. Given the relationship between the peroxisomal redox state and overall cellular redox balance, we investigated the role of GDAP1 in peroxisomal function and mitochondrial MCSs maintenance by using high-resolution microscopy, live cell imaging with pH-sensitive fluorescent probes, and transcriptomic and lipidomic analyses in the *Gdap1*^−/−^ mice and patient-derived fibroblasts.

We demonstrate that GDAP1 deficiency disrupts mitochondria-peroxisome MCSs and leads to peroxisomal abnormalities, which are reversible upon pharmacological activation of PPARγ or glutathione supplementation. These results identify GDAP1 as a new tether of mitochondria-peroxisome MCSs that maintain peroxisomal number and integrity. The supply of glutathione (GSH-MEE) or GDAP1 overexpression suffices to rescue these MCSs. Furthermore, GDAP1 may regulate the redox state within the microdomain of mitochondrial MCSs, as suggested by decreased pH at mitochondria-lysosome contacts in patient-derived fibroblasts, highlighting the relationship between GDAP1 and redox-sensitive targets.

Finally, *in vivo* analysis of sciatic nerve tissue in *Gdap1*^−/−^ mice revealed significant axonal structural abnormalities, including nodes of Ranvier disruption and defects in the distribution and morphology of mitochondria, lysosomes, and peroxisomes, emphasizing the importance of GDAP1 in sustaining axon integrity in the peripheral nervous system. Taken together, this study positions GDAP1 as a multifunctional protein that mediates mitochondrial interaction with cellular organelles of diverse functions, contributes to redox state sensing, and helps maintain axonal homeostasis. In addition, we identify PPAR as a novel therapeutic target, based on knowledge of the underlying pathogenetic mechanisms.

## Introduction

Pathogenic variants in the Ganglioside-induced differentiation-associated protein 1 gene (*GDAP1)* cause Charcot-Marie-Tooth (CMT) disease^1,2^, an axonal neuropathy^1,3^ that may present some demyelinating lesions^4^. GDAP1 is an integral protein of the outer mitochondrial membrane (OMM)^5–7^ classically described as a regulator of mitochondrial dynamics, participating in both mitochondrial fission^8^ and mitochondrial transport along the axon^5,6,9–11^. Another role of this protein is its involvement in mitochondrial membrane contact sites (MCSs) with the plasma membrane^11,12^, the endoplasmic reticulum (ER) at the mitochondria-associated membranes (MAMs)^9,11^ and the lysosomes^9,13^. Moreover, GDAP1 deficiency reduces these contacts, impairing their functionality^9–11,13^. These findings show that GDAP1 is crucial in regulating the positioning of these organelles, although its precise role in their biogenesis and functional processes remains poorly understood.

GDAP1 belongs to an atypical glutathione S-transferase (GST) family of proteins^14^. While the certainty of GDAP1 GST activity is debated^15,16^, it has been reported to exhibit glutathione-conjugating and membrane-remodeling activities^16^. GDAP1 deficiency increases oxidative stress in the peripheral nervous system^17^ and patient-derived fibroblasts^18^, along with decreased cellular glutathione (GSH) levels^9,19^, the primary redox buffer^20^. All these data about GDAP1 point to its possible function as a cellular redox sensor^15^. However, whether GDAP1 acts as a sensor for the redox state at mitochondrial MCSs is entirely unknown. Additionally, it has been proposed that GDAP1 is also located in peroxisomes, but its possible role in mitochondria–peroxisomes MCSs has not been investigated yet^21^.

MCSs shape extensive inter-organelle networks for cellular function and tissue homeostasis^22,23^. They serve as platforms for lipid synthesis, calcium signaling, and mitochondrial function by facilitating lipid and ion exchange between cellular compartments. Therefore, disruptions in pH balance at these contact sites could lead to significant cellular dysfunction. Proper pH balance is crucial for the enzymes involved in lipid metabolism and membrane transport, as pH gradients and local variations can significantly impact the structure and dynamics of molecular assemblies like bilayer membranes^24^.

This study proposes that GDAP1 plays a role in maintaining the redox balance within cells by interacting with peroxisomes and within the mitochondrial MCSs. In cells, we have shown that GDAP1 deficiency leads to peroxisomal abnormalities, which can be fully restored through pharmacological activation of PPARγ. Additionally, GDAP1 acts as a tether of mitochondria-peroxisomes MCSs, and restoration of glutathione or overexpression of GDAP1 successfully rescues these connections when *GDAP1* is mutated. In mitochondria-lysosome MCSs, the observed decrease in pH in GDAP1-deficient cells suggests that this protein has a redox-sensing function within these microdomains. Moreover, GDAP1 deficiency results in significant structural and organelles abnormalities in sciatic nerve axons. Overall, our results demonstrate that GDAP1 is essential for peroxisomes and mitochondrial MCSs functions in both the soma and axon of neural cells.

## Results

### GDAP1 is a tether of mitochondria–peroxisome MCSs

**Figure 1.**
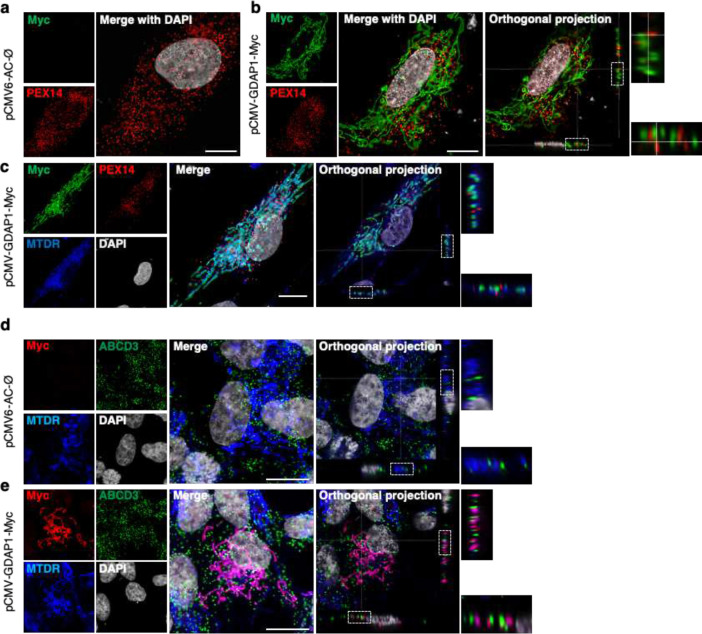
GDAP1 is not located in the peroxisomal membrane. **a**, GDAP1-Myc and PEX14 staining in control fibroblasts transfected with pCMV-AC-Ø plasmid. Scale bar 10 μm. **b**, GDAP1-Myc and PEX14 staining in control fibroblasts transfected with pCMV-GDAP1-Myc plasmid. Orthogonal projections and magnification details are shown. Scale bar 10 μm. **c**, GDAP1-Myc, PEX14 and MitoTracker Deep Red (MTDR) staining in control fibroblasts transfected with pCMV-GDAP1-Myc plasmid. Orthogonal projections and magnification details are shown. Scale bar 10 μm. **d,** GDAP1-Myc and ABCD3 staining in SH-SY5Y neuroblastoma cells transfected with pCMV-AC-Ø plasmid. Orthogonal projections and magnification details are shown. Scale bar 10 μm. **e,** GDAP1-Myc and ABCD3 staining in SH-SY5Y cells transfected with pCMV-GDAP1-Myc plasmid. Orthogonal projections and magnification details are shown. Scale bar 10 μm.

We first examine, by high-resolution confocal microscopy, GDAP1 localization in the peroxisomes. Control fibroblasts were transfected either with an empty vector (pCMV-AC-Ø) (Fig. 1a) or with wild-type GDAP1 in fusion with Myc (pCMV-GDAP1-MYC) (Fig. 1b, c). Mitochondria and peroxisomes were stained using the MitoTracker Deep Red (MTDR) probe and α-Peroxin 14 (PEX14, a peroxisomal transmembrane protein), respectively, and GDAP1 with α-Myc. A parallel analysis was conducted in the human neuroblastoma cell line SH-SY5Y, wherein peroxisomes were stained with α-ABCD3 (ATP Binding Cassette Subfamily D Member 3) (Fig. 1d, e). Our observations consistently showed that GDAP1 is exclusively located on the OMM and colocalizes with the mitochondrial marker MTDR. Moreover, orthogonal projections revealed contact between mitochondrial and peroxisomes, suggesting that GDAP1 may play a role in these interactions.

**Figure 2.**
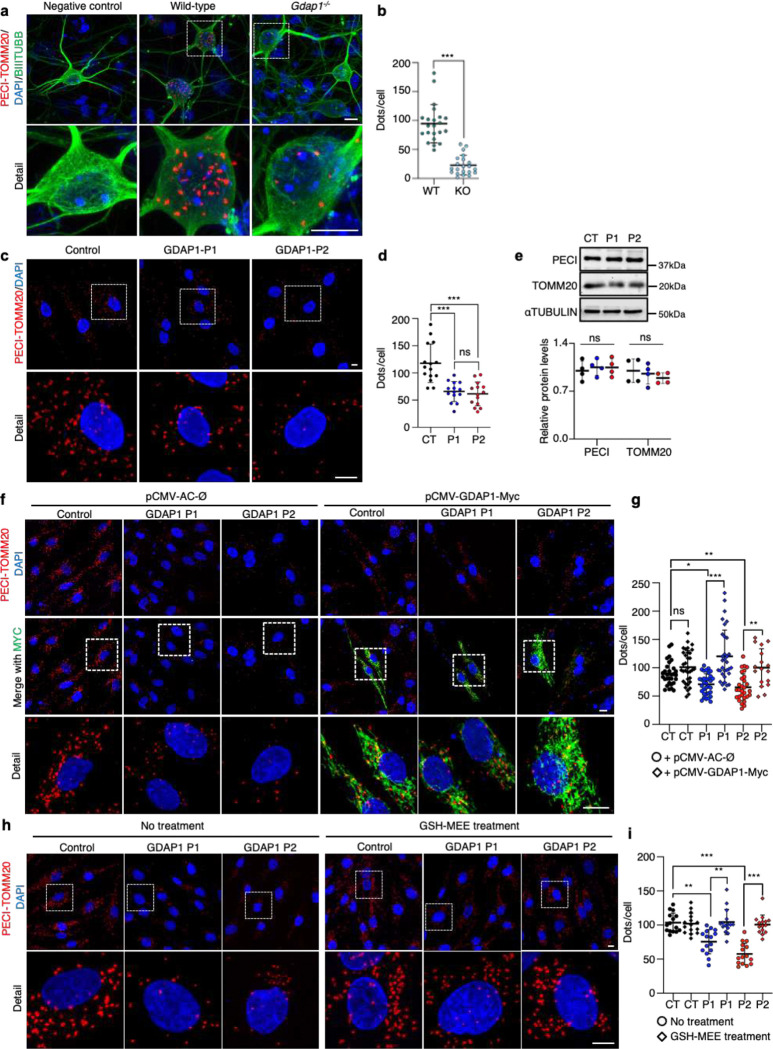
GDAP1 is a tether of mitochondria—peroxisome membrane contact sites. **a**, PLA assay between endogenous PECI and TOMM20 in control and *Gdap1*^−/−^ eMNs. BIIITUBB is also stained. A detail is shown. Scale bar: 10 μm. **b**, Quantification of the number of PECI-TOMM20 dots per neuron. Mann-Whitney test (WT=22, KO=21, neurons, three independent cultures). **c**, PLA assay between endogenous PECI and TOMM20 in control and GDAP1 patients' fibroblasts. A detail is shown. Scale bar: 10 μm. **d**, Quantification of the number of PECI-TOMM20 dots per cell. Kruskal-Wallis followed by Dunn's multiple comparisons test. (CT=64, P1=70, P2=61 fibroblasts, three independent experiments). **e**, Western blot analysis of PECI and TOMM20 proteins in control and GDAP1 patients' fibroblasts. Quantification is shown in the panel below. One sample *t*-test (four independent experiments). **f**, PLA assay between endogenous PECI and TOMM20 in control and GDAP1 patients’ fibroblasts transfected with pCMV-AC-Ø or pCMV-GDAP1-Myc. A detail is shown. Scale bar: 10 μm. **g**, Quantification of the number of PECI-TOMM20 dots per cell. Kruskal-Wallis followed by Dunn's multiple comparisons test. (CT=30, P1=33, P2=29, CT+GDAP1=34, P1+GDAP1=32, P2+GDAP1=17 fibroblasts, four independent experiments). **h**, PLA assay between PECI and TOMM20 in untreated control and GDAP1 patients' fibroblasts or after GSH-MEE treatment. A detail is shown. Scale bar: 10 μm. **i,** Quantification of the number of PECI-TOMM20 dots per cell. Kruskal-Wallis followed by Dunn's multiple comparisons test. (CT=15, P1=15, P2=15, CT+GSH=15, P1+GSH=15, P2+GSH=15 fibroblasts, three independent experiments). All quantitative data are presented as mean ± SD and individual values are displayed as dots. For all comparisons, *p<0.05, **p<0.01, ***p<0.001, ns: not significant.

Peroxisomes and mitochondria are closely intertwined, sharing division machinery proteins, including GDAP1^21,25^. Considering the GDAP1 role in peroxisomal fission and morphology^21^, we aimed to delve deeper into its potential involvement in mitochondria–peroxisome contacts. For this purpose, we analyzed the interaction of TOMM20 (translocase of the mitochondrial outer membrane complex subunit 20 of mitochondria) and PECI (acyl-coenzyme A-binding domain (ACBD)2/ECI2 isoform A of peroxisomes), the unique tethering complex for mitochondria–peroxisome MCSs in mammals^26^. Proximity ligation assay (PLA) experiments in *Gdap1*^−/−^ cultured embryonic motor neurons (eMNs) revealed a pronounced reduction in TOMM20–PECI interaction in the absence of Gdap1 (Fig. 2a–b). Since this result suggested that GDAP1 participates in mitochondria–peroxisomes MCSs, we investigated TOMM20–PECI interaction in fibroblasts from two CMT-*GDAP1* patients. Patient 1 (P1) is a homozygous carrier of the missense variant (*GDAP1*:p.Trp67Leu) in the amino glutathione-S-transferase (GST) domain. Patient 2 (P2), described previously^25^, is a compound heterozygous of two loss-of-function pathogenic variants (the nonsense variant in the α4-α5 loop (*GDAP1*:p.Gln163X) and a frameshift variant in the carboxyl-terminal domain (*GDAP1*:p.Thr288NfsX3) (Supplementary Fig. 1a–c). We found that the TOMM20–PECI interaction was significantly reduced in both patients (Fig. 2c, d). This reduction did not occur because of lower TOMM20 or PECI expression, as shown by Western blot (Fig. 2e). Furthermore, overexpression of wild-type GDAP1 in the patients' fibroblasts rescued the number of TOMM20–PECI interaction (Fig. 2f, g). Therefore, GDAP1 is a tether between the mitochondrial and peroxisomal membranes rather than a peroxisomal protein.

Peroxisomes play a crucial role in cellular metabolism and in the regulation of redox balance^27,28^ and GDAP1 deficiency increases oxidative stress^17–19^. Therefore, we also examined mitochondria–peroxisome contacts in the patients' fibroblasts treated with the antioxidant glutathione monoethyl ester (GSH-MEE). After the treatment, the TOMM20–PECI interaction raised to levels observed in the control fibroblast (Fig. 2h, i). This result suggests that the pathophysiology caused by GDAP1 deficiency is linked to the dysfunction of its GST domains.

### GDAP1 deficiency causes abnormalities in the number and morphology of peroxisomes that preserve their enzymatic capacity

**Figure 3.**
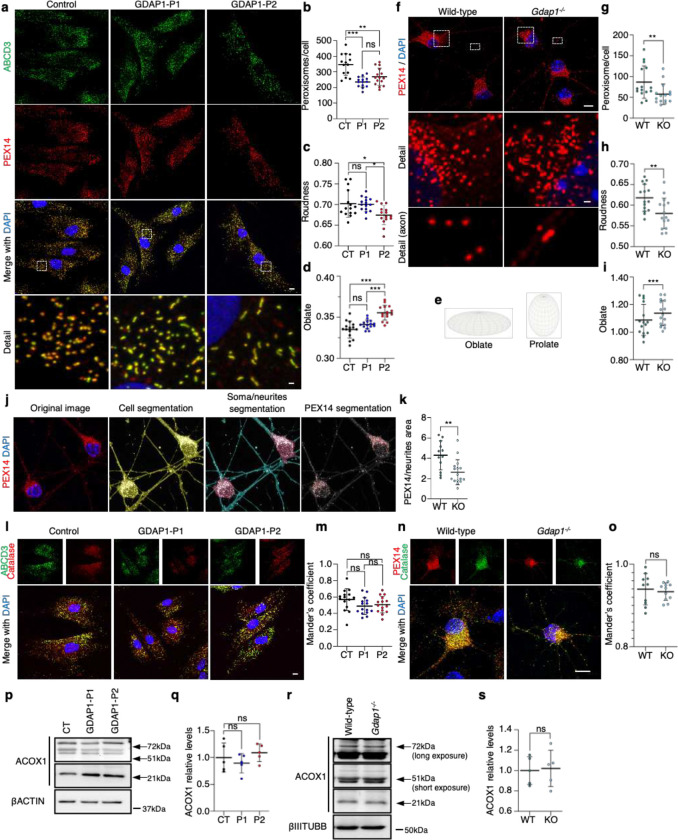
GDAP1 deficiency leads to abnormalities in the number and morphology of peroxisomes without affecting their enzymatic capacity. **a**, Representative images of peroxisomal membrane proteins ABCD3 and PEX14, and DAPI in control and GDAP1 patients’ fibroblasts. A magnification is shown. Scale bar 10 μm, detail 1 μm. **b**, Peroxisomal roundness quantification. One-way ANOVA followed by Tukey's post hoc. (CT=47, P1=58, P2=61 fibroblasts, three independent experiments). **c**, Peroxisome number per cell. One-way ANOVA followed by Tukey's post hoc. (CT=47, P1=58, P2=61 fibroblasts, three independent experiments). **d**, Oblate shape descriptor quantification. One-way ANOVA followed by Tukey's post hoc. (CT=47, P1=58, P2=61 fibroblasts, three independent experiments). **e**, Representation of oblate and prolate shape descriptors. **f**, Representative images of peroxisomal membrane protein PEX14, and DAPI in cultured eMNs from wild-type and *Gdap1*^−/−^ mice. General and axonal details are shown. Scale bar 10 μm, detail 1 μm. **g**, Quantification of peroxisomal roundness. Mann-Whitney test. (WT=31, KO=31 neurons, three independent primary cultures). **h**, Peroxisome number per neuron. Mann-Whitney test. (WT=31, KO=31 neurons, three independent primary cultures). **i**, Quantification of oblate shape descriptor. One-sample *t*-test. (WT=31, KO=31 neurons, three independent primary cultures). **j**, Image processing and segmentation for soma/neurites peroxisome number analysis. **k**, Quantification of peroxisome number in neurites of eMNs from wild-type and *Gdap1*^−/−^ mice. Mann-Whitney test. (WT=31, KO=31 neurons, three independent primary cultures). **l**, Representative images of ABCD3 and Catalase in control and GDAP1 patients' fibroblasts. Scale bar 10μm. **m,** Mander’s coefficient quantification. One-way ANOVA followed by Tukey's post hoc. (n=15 ROI, from three independent experiments). **n,** Representative images of PEX14 and Catalase in cultured eMNs from wild-type and *Gdap1*^−/−^ mice. Scale bar 10μm. **o,** Mander’s coefficient quantification. One-sample *t* test. (WT=10, KO=11 ROI, three independent primary cultures). **p,** Western blot analysis of ACOX1 isoforms in GDAP1 patients' fibroblasts. **q,** Quantification of ACOX1 relative levels in GDAP1 patients’ fibroblasts. One-Sample *t* test. (five independent experiments). **r,** Western blot analysis of ACOX1 isoforms in eMNs from wild-type and *Gdap1*^−/−^ mice. **s,** Quantification of ACOX1 relative levels in eMNs. One-sample *t* test. (five independent primary cultures). All quantitative data are presented as mean ± SD and individual values are displayed as dots. For all comparisons, *p<0.05, **p<0.01, ***p<0.001, ns: not significant. ROI: region of interest.

Previous studies have shown that CMT-*GDAP1* missense variants in the N-terminal domain exclusively affect mitochondrial dynamics. In contrast, the loss or C-terminal truncation of GDAP1 additionally affects peroxisomal dynamics^21^ (Supplementary Fig. 1). To further investigate how the deficiency of GDAP1 affects peroxisomes, we quantified and analyzed the peroxisome morphology in the patients' fibroblasts by immunostaining with α-ABCD3 and α-PEX14^29^ (Fig. 3a). The patients' fibroblasts exhibited a reduced number of peroxisomes per cell compared to control fibroblasts (Fig. 3b). Qualitatively the Patient 1 peroxisomes were similar to the control. In contrast, Patient 2 showed elongated peroxisomes, as indicated by a decrease in roundness (Fig. 3c), along with an increase in the oblate shape descriptor (Fig. 3d), which is defined as a spheroid formed by revolving an ellipse around its minor axis (Fig. 3e). We also analyzed peroxisome number and morphology in cultured eMNs from *Gdap1*^−/−^ mice (Fig. 3f). PEX14 staining revealed in *Gdap1*^−/−^ eMNs a significant reduction in the number of peroxisomes and an elongated morphology characterized by an increase in the oblate shape descriptor (Fig. 3g–i). To investigate whether peroxisomes are defective in neurites-axon structures, we employed an automated soma/neurites segmentation (Fig. 3j; Supplementary Fig. 2). We detected a diminished number of peroxisomes in neurites-axon from *Gdap1*^−/−^ eMNs compared to wild-type eMNs (Fig. 3k).

To investigate the potential consequences of peroxisomal morphological alterations on their enzymatic capacity, we initially assessed the presence or absence of import-competent peroxisomes and peroxisomal membranes by immunostaining the Catalase^30^. We found no Catalase mislocalization in patients' fibroblasts (Fig. 3l, m) or in *Gdap1*^−/−^ eMNs (Fig. 3n, o), confirming the correct localization of this enzyme inside peroxisomes. Subsequently, we examined the cleavage of Acyl-CoA oxidase 1 (ACOX1), an enzyme that catalyzes the rate-limiting reaction for lipids beta-oxidation in peroxisomes^30^. We observed cleaved ACOX1 isoforms (51kDa and 21kDa) in both GDAP1 patients' fibroblasts (Fig. 3p, q) and *Gdap1*^−/−^ eMNs (Fig. 3r, s), indicating the correct processing of this enzyme within the beta-oxidation pathway. These findings argued that GDAP1 deficiency decreases the number of peroxisomes and alters their morphology without affecting their enzymatic capacity.

### GDAP1 deficiency lowers the pH at mitochondrial membrane contact sites

**Figure 4.**
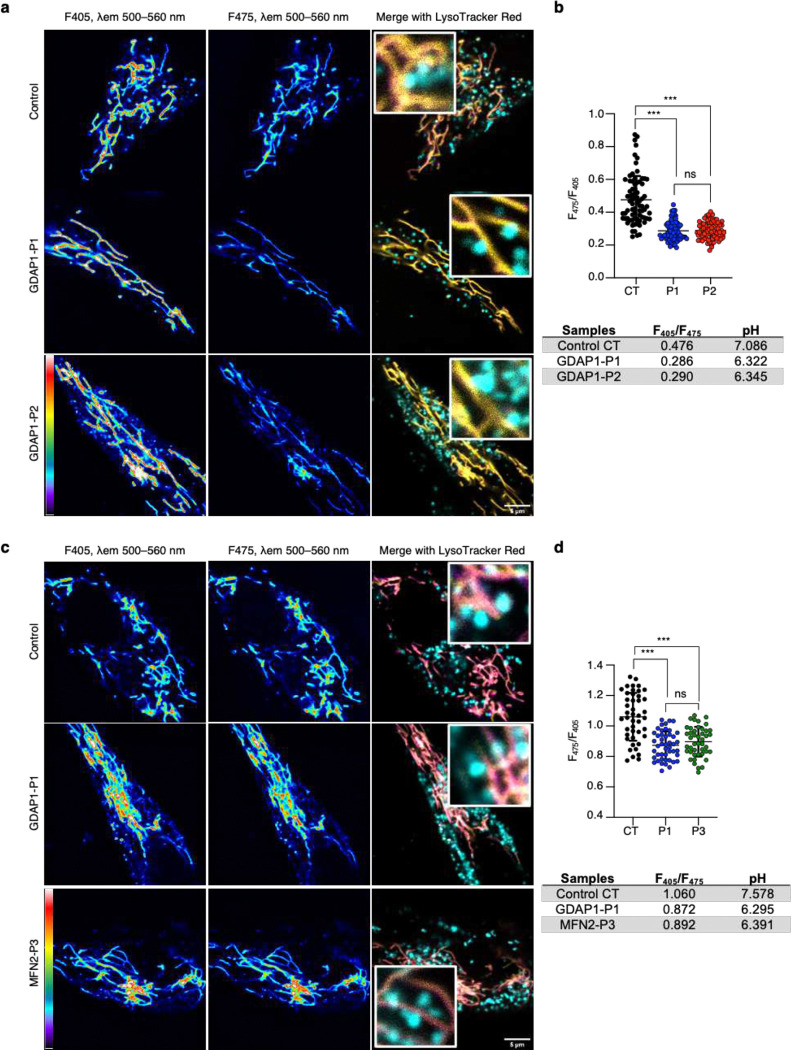
GDAP1 deficiency lowers the pH at mitochondrial membrane contact sites. **a**, Representative confocal microscopy images of control and GDAP1 patients' fibroblasts transfected with TOMM20-RpHluorin2 and stained with Lysotracker Red. Pseudocolored images display the pH indicator signal in two channels (F_405_/F_475_). Fluorescent images (λ_em_ 500–560 nm) were detected with excitation at λ_ex_ 405 nm and λ_ex_ 475 nm. The pseudocolored scale is shown at the bottom left. Warm colors such as white and red represent maximum intensities, whereas cold colors like blue are representative of low intensities. Scale bar: 5 *μ*m. **b**, pH quantification in mitochondria-lysosome contacts by F_475_/F_405_ ratio in control and GDAP1 patients’ fibroblasts. Kruskal-Wallis followed by Dunn's multiple comparisons test (n=75–80 events/sample). Data are presented as mean ± SD, and individual values are displayed as dots. ***p<0.001, ns: not significant. **c**, Representative confocal microscopy images of control, GDAP1 and MFN2 patients' fibroblasts transfected with TOMM20-RpHluorin2 and stained with Lysotracker Red. Pseudocolored images display the pH indicator signal in two channels (F_405_/F_475_). Fluorescent images (λ_em_ 500–560 nm) were detected with excitation at λ_ex_ 405 nm and λ_ex_ 475 nm. The pseudocolored scale is shown at the bottom left. Warm colors such as white and red represent maximum intensities, whereas cold colors like blue are representative of low intensities. Scale bar: 5 *μ*m. **d**, pH quantification in mitochondria-lysosome contacts by F_475_/F_405_ ratio in control, GDAP1 and MFN2 patients’ fibroblasts. Kruskal-Wallis followed by Dunn's multiple comparisons test (n=44–46 events/sample). All quantitative data are presented as mean ± SD, and individual values are displayed as dots. ***p<0.001, ns: not significant.

Given the GDAP1 relationship to peroxisomes^21^, the redox state^9,15,17,18^, and the mitochondrial MCSs^9,11–13^, we wondered if GDAP1 plays a role in the redox state of the mitochondrial MCSs microenvironment. We investigated the redox status within mitochondria–lysosome MCSs that we previously characterized in detail ^9,13,31^. We measured the pH *in vivo* of these microdomains, as changes in pH and redox status are interdependent processes in the cells^20^. We used the ratiometric pH-sensitive probe pHluorin2 (refer to methods)^32^. Initially, we established a calibration curve employing the GPI-pHluorin2 construct, which expresses a Glycosylphosphatidylinositol that localizes to the plasma membrane (Supplementary Fig. 3a, b). To measure pH at mitochondria–lysosome MCSs, we cloned the outer mitochondrial membrane protein TOMM20 into a pHluorin2 vector to obtain the TOMM20-pHluorin2 construct. In the transfected fibroblasts, we visualized lysosomes with the Lysotracker Red probe to measure the pH of mitochondria–lysosome MCSs accurately. We found that control fibroblasts exhibited a physiological pH (7.086), while Patient 1 and Patient 2 showed significant reductions, with pH values recorded at 6.322 and 6.345, respectively (Fig. 4a, b).

To further investigate the role of GDAP1 in maintaining redox balance within mitochondrial MCSs, we conducted the same experiment by transfecting TOMM20-pHluorin2 into fibroblasts from Patient 1 and another CMT patient (P3) who carries a heterozygous p.Arg104Trp variant in the *MFN2* gene for the OMM Mitofusin 2. We previously showed that MFN2 interacts with GDAP1, is located in mitochondria–lysosome MCSs, and MFN2^Arg104Trp/+^ fibroblasts showed a reduction in these contacts, preserving the lysosomal morphology^31^. In transfected Patient 3 fibroblasts, we recorded a pH of 6.391, a decrease in pH compared to the control (Fig. 4c, d). Interestingly, we also detected a marked reduction in GDAP1–MFN2 interaction in these fibroblasts by PLA (Supplementary Fig. 3c, d), indicating a potential dysfunction of GDAP1 in these cells. These results show that GDAP1 maintains the pH of mitochondria–lysosomes MCSs microdomains. They also suggest the redox status as a key element in the MCSs pathophysiology of CMT axonopathy.

### GDAP1 deficiency disrupts phospholipid profiles and modulates gene expression associated with cellular stress responses

**Figure 5.**
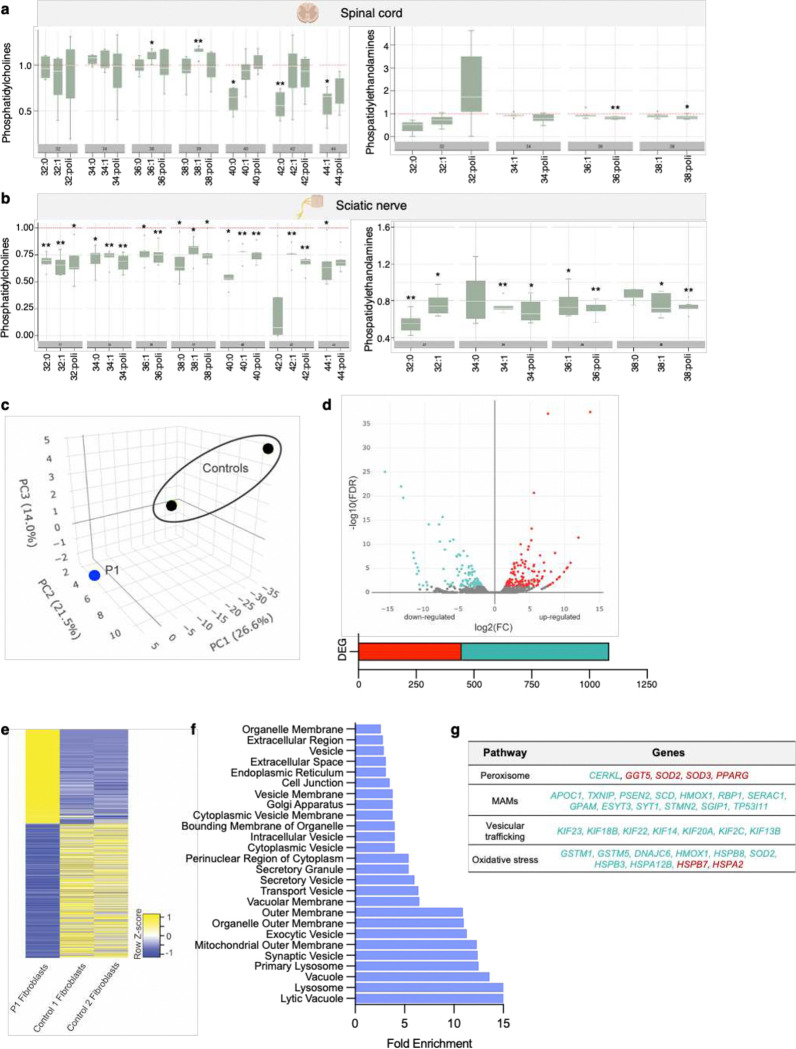
GDAP1 deficiency disrupts phospholipid profiles and modulates gene expression associated with cellular stress responses. **a**, Fold-change ratio quantification of phosphatidylcholines (left) and phosphatidylethanolamines (right) in the spinal cord from wild type and *Gdap1*^−/−^ mice by UHPLC-TOF. (5 animals/genotype). **b**, Fold-change ratio quantification of phosphatidylcholines (left) and phosphatidylethanolamines (right) sciatic nerve from wild type and *Gdap1*^−/−^ mice by UHPLC-TOF. (5 animals/genotype). Two-sided one-sample *t*-test. The box plot lines correspond from the bottom of the box to the top: 25th percentile, median percentile, 75th percentile. The whiskers extend to the minimum and maximum values. *P<0.05; **P<0.01. **c,** PCA plot shows the distribution of DEG in the patient (P1) sample as compared to two control fibroblasts lines. Further, the 1086 DEGs are fairly evenly distributed between up and downregulated categories as illustrated in the **d,** volcano plot and **e,** heatmap. The **e,** biological pathway enrichment reveals 19 pathways with 3 to 24 fold enrichment, all which have a q-value <2.5^E−3^. **g,** summary of the specific pathways with the upregulated and downregulated genes identified in the RNAseq analysis.

Since Charcot-Marie-Tooth disease is peripheral neuropathy, we evaluated indicative parameters of oxidative stress in spinal cords and sciatic nerves of six-month-old wild-type and *Gdap1*^−/−^ mice. Specifically, we assessed lipid peroxidation in these samples using Ultra-High Performance Liquid Chromatography combined with Time-of-Flight Mass Spectrometry (UHPLC-TOF). We measured phosphatidylcholine (PC) and phosphatidylethanolamine (PE), the two major OMM phospholipids^33^. *Gdap1*^−/−^ to wild-type ratio for each lipid indicates lipid biosynthesis and metabolism levels (Fig. 5a–b). The *Gdap1*^−/−^/wild-type ratio in the spinal cord was predominantly close to 1, indicating no differences between samples (Fig. 5a). In contrast, we observed a significant reduction in the *Gdap1*^−/−^/wild-type ratio of both phospholipids in the sciatic nerve (Fig. 5b). This observation suggests a decrease in biosynthesis or an increase in lipid metabolism due to a rise in membrane oxidation in *Gdap1*^−/−^ mice. All these results show that the lack of GDAP1 alters the oxidative status and redox balance in peripheral nerves.

To gain more insight into the impact of GDAP1 deficiency on redox status, we conducted a transcriptomic analysis in GDAP1 Patient 1 fibroblasts. Principal component analysis (PCA) separated the control samples from the patient sample, with no outliers detected (Fig. 5c). This analysis identified 1086 differentially expressed genes (DEG) in this patient, including 447 upregulated genes and 639 downregulated genes, compared to control fibroblasts (Fig. 5d, e). Pathway enrichment revealed dysregulation in several gene expression networks, notably those involved in peroxisomal biology (*GGT5*, *SOD2*, *SOD3*, *CERK*, and *PPARγ*), organelle-to-organelle contacts such as MAMs (*APOC1*, *TXNIP*, *PSEN2*, *SCD*, *HMOX1*, *RBP1*, *SERAC1*, *GPAM*, *ESYT3*, *SYT1*, *STMN2*, *SGIP1*, *TP53I11*), vesicular trafficking (*KIF23*, *KIF18B*, *KIF22*, *KIF14*, *KIF20A*, *KIF2C*, and *KIF13B*), and oxidative stress sensoring (Fig. 5f, g).

Regarding the oxidative stress sensing pathways, the RNA-seq data highlighted the altered expression of genes linked to cellular stress responses. Notably, *HSPB7* and *HSPA2*, both involved in skeletal muscle stress response, were upregulated, underscoring their potential roles in the stress response mechanisms in GDAP1 deficiency^34,35^. On the other hand, we identified the downregulation of *GSTM1, GSTM5, DNAJC6, HMOX1, HSPB8, SOD2, HSPB3*, and *HSPA12B*, which are involved in antioxidant defense. *GSTM1* and *GSTM5*, which encode enzymes involved in cell detoxification^36^, showed reduced expression levels. Additionally, decreased expression of *DNAJC6*, a co-chaperone involved in protein folding^37^, suggests disruptions in protein homeostasis and stress responses. *HMOX1*, a crucial gene for heme metabolism and antioxidant defense^38^, also showed reduced expression, indicating impaired protection against oxidative damage. Further downregulation of *HSPB8, SOD2, HSPB3*, and *HSPA12B* reflects weakened cellular stress responses^39–41^. *HSPB8* assists in protein folding, and its decreased expression may compromise the cell's ability to handle stress. *SOD2*, an important antioxidant enzyme, also exhibited reduced expression, implying a diminished defense against oxidative damage. *HSPB3* protects cells from stress-induced apoptosis, and *HSPA12B*, another stress-related protein, also showed reduced levels, indicating compromised stress adaptation.

Our lipidomics in the *Gdap1*^−/−^ mice and transcriptomics in Patient 1 fibroblasts argued that GDAP1 deficiency reduces antioxidant defenses and resilience to stress, leading to a general dysfunction of the redox state, which would contribute to the *GDAP1*-related pathophysiology.

### PPARγ activation restores the peroxisomal defects in GDAP1-deficient fibroblasts

Peroxisome proliferator-activated receptor gamma (PPARγ) is a nuclear receptor functioning as a transcription factor for several genes, including the E3 ubiquitin ligase membrane-associated RING-CH-type finger 5 (MARCH5)^42^. This E3 ubiquitin ligase is central for mitochondrial-dependent peroxisome biogenesis^43^. Since we found in the Patient 1 fibroblasts that *PPARγ* is downregulated (Fig. 5g) and the peroxisomal population significantly decreases when *GDAP1* is defective (Fig. 3a), we hypothesized that PPARγ activation with an agonist could rescue the peroxisomal defects found in the patients.

**Figure 6.**
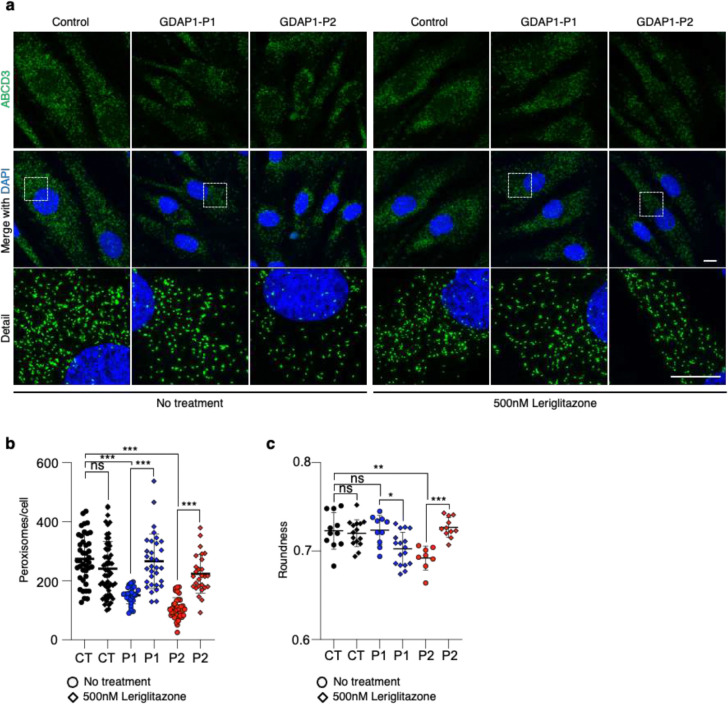
The PPARγ agonist Leriglitazone restores peroxisomal defects in GDAP1 fibroblasts. **a,** Representative images of peroxisomal membrane protein ABCD3, and DAPI in untreated control and GDAP1 patients' fibroblasts, or after Leriglitazone treatment at 500nM during 48 hours. A magnification is shown. Scale bar 10 μm. **b**, Peroxisome number per cell. One-way ANOVA followed by Tukey's post hoc. (CT=42, P1=27, P2=38, CT+Leri=61, P1+Leri=32, P2+Leri=27 fibroblasts, two independent experiments). **c**, Peroxisomal roundness quantification. One-way ANOVA followed by Tukey's post hoc. (CT=6769, P1=4711, P2=4061, CT+Leri=7146, P1+Leri=8515, P2+Leri=6029 peroxisomes, two independent experiments). All quantitative data are presented as mean ± SD and individual values are displayed as dots. For all comparisons, *p<0.05, **p<0.01, ***p<0.001, ns: not significant.

To assess this hypothesis, we treated for 48 hours the patients' fibroblasts with 500 nM of Leriglitazone (Fig. 6), a novel PPARγ agonist currently under development for treating nervous system disorders^44^. After treatment, we immunolabeled the peroxisomes with α-ABCD3 to quantify the peroxisomal population (a metric disrupted in both patients) and morphology (specifically altered in Patient 2). The treatment fully rescues the peroxisomal population in both patients (Fig. 6b), which reached the control levels. Besides, we found differences between patients related to residual GDAP1 expression. Patient 1 expressed lower levels of GDAP1^p.Trp67Leu^ (Supplementary Fig. 1d) showed elongated peroxisomes, suggesting an effect of the mutant peptide upon restored peroxisomes biogenesis compared to controls. Strikingly, we found that in Patient 2 fibroblasts lacking GDAP1 (Supplementary Fig. 1d), the Leriglitazone treatment successfully restored normal peroxisomal morphology (Fig. 6c). These results suggest that GDAP1 is involved in peroxisome biogenesis, contributing not only to organelle fission but also to mitochondrial membrane-dependent biogenesis.

### Mitochondria, lysosomes, and peroxisomes are defective in the sciatic nerves of *Gdap1*^−/−^ mice

**Figure 7.**
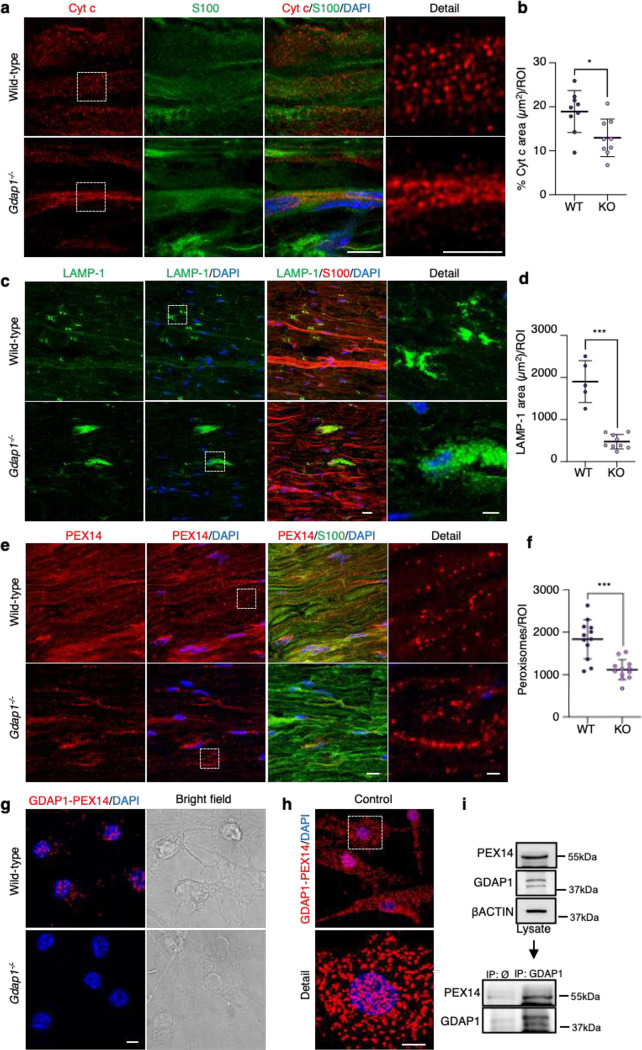
Mitochondria, lysosomes and peroxisomes are disrupted in sciatic nerves of *Gdap1*^−/−^ *mice.* **a**, Representative images of Cytochrome c and S100 staining in sciatic nerve sections from wild-type and *Gdap1*^−/−^ mice. Magnification details are shown. Scale bar 8 μm, detail 4 μm. **b**, Quantification of Cytochrome c percentage area per ROI. Mann-Whitney test. (n=9 WT and n=9 KO ROIs, from three animals/genotype). **c**, Representative images of LAMP-1 and S100 staining in sciatic nerve sections from wild-type and *Gdap1*^−/−^ mice. Magnification details are shown. Scale bar 15 μm, detail 5 μm. **d**, Quantification of LAMP-1 total area per ROI. Mann-Whitney test. (n=5 WT and n=9 KO ROIs, from three and five animals/genotype, respectively). **e**, Representative images of PEX14 and S100 staining in sciatic nerve sections from wild-type and *Gdap1*^−/−^ mice. Magnification details are shown. Scale bar 10 μm, detail 2 μm. **f**, Quantification of peroxisome number per ROI. Mann-Whitney test. (n=12 ROI, from three animals/genotype). **g**, Proximity ligation assay (PLA) between endogenous GDAP1 and PEX14 in control and *Gdap1*^−/−^ eMNs. Bright field is shown. Scale bar: 10 μm. **h**, PLA assay between endogenous GDAP1 and PEX14 in control fibroblasts. A detail is shown. Scale bar: 10 μm. **i**, Co-IP assay of endogenous GDAP1 and PEX14 in control fibroblasts. All quantitative data are presented as mean ± SD and individual values are displayed as dots. For all comparisons, *p<0.05, **p<0.01, ***p<0.001, ns: not significant. ROI: region of interest.

Next, we analyzed the localization and distribution of mitochondria, lysosomes, and peroxisomes in the sciatic nerve of *Gdap1*^−/−^ mice labeled with specific markers. All the organelles examined exhibited an abnormal distribution within the sciatic nerve axons. Mitochondria showed a clustered distribution (Fig. 7a) and a decrease in the percentage of the occupied area (Fig. 7b). Lysosomes, which show a symmetrical arrangement in the paranodal area in the wild-type sciatic nerves, presented morphological abnormalities in the *Gdap1*^−/−^ sciatic nerves. Moreover, the absence of Gdap1 cause giant lysosomes around Schwann cells nuclei and their significant reduction in the paranodal area (Fig. 7c, d). Regarding peroxisomes, *Gdap1*^−/−^ sciatic nerves showed reduced number, abnormal distribution, and aggregation compared to wild-type mice (Fig. 7e, f). Furthermore, this peroxisomal distribution resembled the abnormal mitochondrial pattern observed in *Gdap1*^−/−^ nerves. Suspecting that the abnormal distribution patterns of both mitochondria and peroxisomes could be related to defects in MCSs interaction between both organelles, we hypothesized that GDAP1 tether with a molecular marker in the peroxisomal membrane. To study the involvement of GDAP1 in mitochondria–peroxisome MCSs, we performed a PLA using α-GDAP1 and α-PEX14. We found the constitutive interaction between GDAP1 and PEX14 in cultured wild-type mouse eMNs (Fig. 7g) and control fibroblasts (Fig. 7h). Co-IP experiments confirmed the GDAP1–PEX14 interaction (Fig. 7i). These findings indicate that the absence of GDAP1 leads to abnormalities in organelles distribution in the peripheral nerves, and highlight the relevance of GDAP1 at mitochondrial MCSs for interaction with other organelles and proper axon physiology.

### Lack of GDAP1 leads to peripheral nerve structural alterations

**Figure 8.**
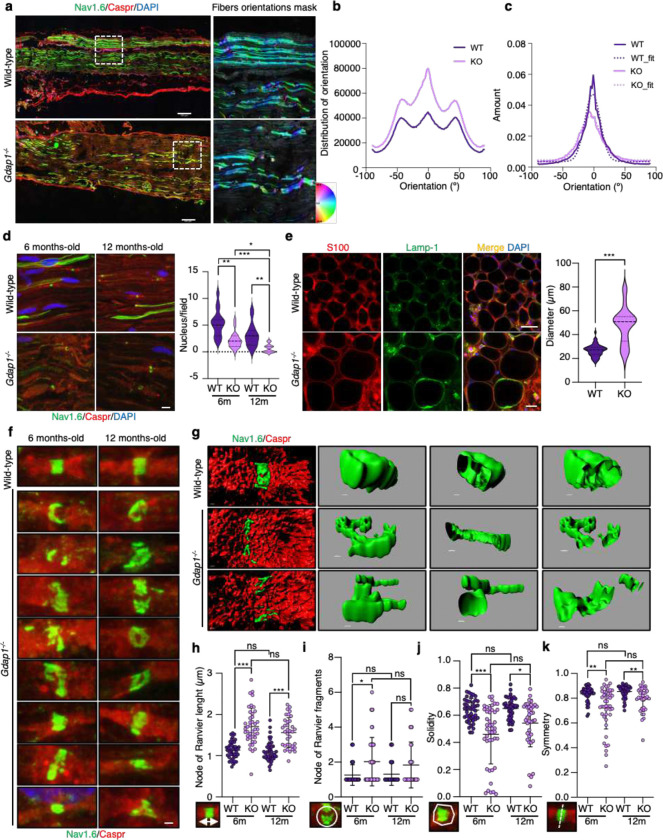
Lack of *Gdap1* causes structural nerve alterations. **a,** Projection of stitched mosaic images depicting the sciatic nerves stained with Nav1.6, Caspr and DAPI from 6-months old wild-type and *Gdap1*^−/−^ mice. On the right, a hue-saturation-brightness (HSB) color map region of Nav1.6 is displayed. These maps are generated using the OrientationJ plugin, where specific orientation angles are assigned to colors and their saturation levels. Scale bar 100 μm. **b-c,** Graphs illustrating the distribution (**b**) and quantity (**c**) of Nav1.6 orientation in wild-type and *Gdap1*^−/−^ nerves. **d**, Representative images of Nav1.6, Caspr and DAPI in sciatic nerve sections from 6-months old and 12-months old wild-type and *Gdap1*^−/−^ mice. Scale bar 10 μm. A violin plot showing the quantification of the number of nuclei per field is shown in the right panel. Kruskal-Wallis followed by Dunn's multiple comparisons test. (n=15 WT-6, n=16 WT-12; n=17 KO-6 and n=18 KO-12 ROIs, from three animals/genotype). **e**, Whole mount staining of sciatic nerves from 6-months old wild-type and *Gdap1*^−/−^ mice. We show S100, Lamp-1 and DAPI of the most distal region, where neuromuscular junctions are stablished. Scale bar 20 μm. A quantification of the axon diameter is shown in the right panel. Studenťs *t*-test (n=50 WT, n=50 KO, axons, respectively). **f,** Representative images of Caspr and Nav1.6 staining in sciatic nerve sections from 6-months old and 12-months old wild-type and *Gdap1*^−/−^ mice. Scale bar 1 μm. **g**, Node of Ranvier 3D projections from 6-months old wild-type and *Gdap1*^−/−^ mice. Different sections and orientations are shown. Scale bar 0.5 μm. **h,** Node of Ranvier length quantification. Kruskal-Wallis followed by Dunn's multiple comparisons test. (n=46 WT-6, n=46 WT-12; n=41 KO-6 and n=35 KO-12 nodes, from three animals/genotype, respectively). **i**, Node of Ranvier fragments quantification. Kruskal-Wallis followed by Dunn's multiple comparisons test. (n=39 WT-6, n=44 WT-12; n=43 KO-6 and n=30 KO-12 nodes, from three animals/genotype, respectively). **j**, Node of Ranvier solidity quantification. Kruskal-Wallis followed by Dunn's multiple comparisons test. (n=45 WT-6, n=45 WT-12; n=44 KO-6 and n=37 KO-12 nodes, from three animals/genotype, respectively). **k**, Node of Ranvier symmetry quantification. Kruskal-Wallis followed by Dunn's multiple comparisons test. (n=45 WT-6, n=45 WT-12; n=44 KO-6 and n=37 KO-12 nodes, from three animals/genotype, respectively). Quantitative data (**h-k**) are presented as mean ± SD and individual values are displayed as dots. For all comparisons, *p<0.05, **p<0.01, ***p<0.001, ns: not significant.

To better define the effects of GDAP1 deficiency on nerves, we conducted a macro morphological analysis of *Gdap1*^−/−^ mice sciatic nerve by mapping the entire structure (Fig. 8a). We applied a filter to detect potential nerve disorganization using an orientation and directional analysis (Fig. 8a right panel). In terms of structure orientation, the applied mask revealed notably higher and distinct peaks in the *Gdap1*^−/−^ nerve, suggesting increased variability in orientation, which appears heterogeneous (Fig. 8b). In terms of directionality, we observed that the wild-type nerve has a narrower peak (center gaussian peak −2.21), reflecting uniformity. However, the *Gdap1*^−/−^ nerve displays a broader peak, slightly shifted to the left (center gaussian peak −5.92), indicating increased variability in the directionality (Fig. 8c). Furthermore, we noted a significant reduction in nuclei number in *Gdap1*-deficient nerves (Fig. 8d), with an even more substantial decrease in 12-month-old mice compared to 6-month-old mice. This change reflects a disruption in Schwann cell–axon communication.

To further study the sciatic nerve structure, we performed a whole mount staining of the mice nerve^45^ (Fig. 8e, left panel). In the *Gdap1*^−/−^ sciatic nerve, we observed disorganized axons with larger diameters in the most distal region, where they connect with the muscles in the posterior compartment of the thigh and with the hamstring portion of the adductor magnus at neuromuscular junctions. These observations correlate with denervation^48^ (Fig. 8e, right graphics).

Since the study of lysosomal distribution in sciatic nerves suggested defects in the nodes of Ranvier (NoR) (Fig. 7c), we investigated their structure. We labeled the sodium channel 1.6 (Nav1.6), a marker of mature nodes^46^, flanked by Caspr-labelled paranodes in the sciatic nerves of 6-months-old and 12-months-old wild-type and *Gdap1*^−/−^ mice. We observed abnormal NoR structures in *Gdap1*^−/−^ sciatic nerves (Fig. 8f), which were confirmed by super-resolution confocal microscopy and 3D projections (Fig. 8g). We quantified morphological and structural NoR parameters by computational image analysis and specific algorithms (see materials and methods, Supplementary Fig. 4). NoR from *Gdap1*^−/−^ mice were longer (Fig. 8h), which was associated with a reduced axon conduction velocity^47^ and they were fragmented (Fig. 8i). We also observed that NoR from *Gdap1*^−/−^ mice had less solidity (Fig. 8j) and symmetry (Fig. 8k), which reflected a disorganized structure. These findings indicate that GDAP1 deficiency impacts overall nerve organization and affects the NoR, potentially contributing to altering the nerve conduction velocity and action potentials as previously seen in electrophysiological studies^48^.

## Discussion

**Figure 9.**
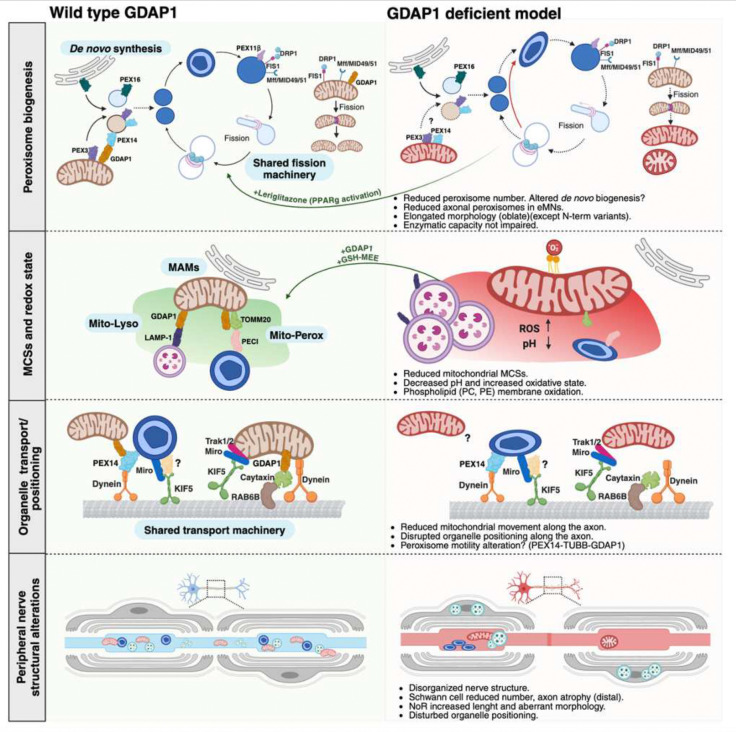
GDAP1 functions and proposed pathophysiological mechanisms in deficient cells. The figure shows the structure of sciatic nerves with nodes of Ranvier, peroxisome biogenesis, which shares machinery with mitochondria, organelle transport along the axon, and mitochondrial-membrane contact sites in wild-type cells (left panel) juxtaposed with *GDAP1* deficient cells (right panel).

Charcot-Marie-Tooth disease involves a complex pathophysiology with multiple genes, pathways, and organelles affecting Schwann cells and axons. Among them*, GDAP1*-related CMT is one of the most frequent recessive axonal neuropathies in many populations^1,2,4,49^. *GDAP1*-related pathophysiology includes reduced mitochondrial MCSs, mitochondrial shape and movement defects within the axon, impaired basal autophagy, and abnormalities in early endosomes and lysosomes^9,10^. Some of these consequences of GDAP1 deficiency can be rescued with treatments that restore the physiological redox state^9^. This study advances the understanding of GDAP1 involvement in redox homeostasis, not only in the soma and axon of neural cells but also in the mitochondrial MCSs (Fig. 9).

GDAP1 participates in the interaction of mitochondria with different organelles at mitochondrial MCSs^9–11,13^. We found that GDAP1 is also involved in mitochondrial-peroxisomal MCSs. GDAP1–PEX14 has been identified as a novel binding partner of these MCSs, positioning GDAP1 as an outer mitochondrial membrane protein that acts as a hub for mediating interactions between mitochondrial MCSs and organelles. Indeed, the absence of GDAP1 disrupts these interactions, which would impair mitochondrial MCSs functionality. Furthermore, GDAP1 deficiency disrupts the redox state within mitochondria–lysosomes MCSs, as evidenced by a significant pH reduction in these compartments. Emerging evidence suggests that GDAP1 may act as a sensor for the cellular redox state, a function that likely relies on its ability to dimerize via the cysteine residue at position 88^50^. In fact, reactive oxygen species (ROS) can modulate proteins at responsive sites, such as cysteines, by inducing conformational changes, such as the formation of disulfide bonds^51^. The dimerization of GDAP1 would enable its function as a sensor of the redox state of mitochondrial MCS, not only with the lysosome membrane but also with other organelles. Another finding supporting the proposal of GDAP1 as a redox sensor for mitochondrial MCSs is that both the overexpression of wild-type GDAP1 and the oxidation-reduction via GSH-MEE treatment restore mitochondria-peroxisome MCSs. We previously found a similar rescue in lysosomes, where GSH-MEE supplementation in the medium promoted physiological proximity^9^. Moreover, ROS could regulate organelle interactions by modifying tethering proteins, possibly altering their conformation and affecting MCS integrity. The ER, mitochondria, and peroxisomes are organelles enriched with redox-related metabolic reactions. Recent studies emphasize the integration of redox signaling within this organelle "triangle," a concept referred to as the MCSs redoxosome^52^. Investigating this regulatory mechanism offers a new direction for our research on GDAP1, which we aim to pursue.

GDAP1 deficiency decreases mitochondria–lysosome contacts, leading to defects in the mitochondrial network, characterized by elongated mitochondria and enlarged lysosomes with abnormal morphology and distribution^9,31^. We now demonstrate a similar effect on peroxisomes that were reduced and showed morphology abnormalities. Moreover, we found a reduction in eMN axonal peroxisomes, a pathological phenotype reported in other neurodegenerative diseases, such as hereditary spastic paraplegia^53^. Reduced peroxisome function in axons can have serious consequences because this organelle plays a role in maintaining myelin sheaths and the function of the nodes of Ranvier^54,55^. Moreover, they are involved in the beta-oxidation of very long-chain fatty acids and the synthesis of plasmalogens, both essential myelin components ^54,55^. Thus, mutations in *GDAP1* can impair peroxisomal function, disrupt lipid metabolism, and contribute to demyelination. Importantly, as occurs in lysosomes, peroxisomal enzyme activity remains unaffected, as evidenced by the proper localization of catalase and the correct cleavage of ACOX1, a limiting beta-oxidation enzyme.

Many aspects of peroxisome biology remain enigmatic, particularly their biogenesis, dynamics, and, notably, their connections to disease and treatments^56^. Our transcriptomics studies indicate that GDAP1 deficiency impairs antioxidant defenses and resilience to stress, which agrees with the oxidative stress consistently reported in *GDAP1* deficiency. Notably, *PPARγ* emerged as the most downregulated gene. This transcription factor regulates the expression of MARCH5 involved in peroxisomal biogenesis^57^. In this work, we treated patients' fibroblasts with the PPARγ agonist Leriglitizone and found full recovery of the peroxisomal population. The implications of this finding are of great clinical significance. This treatment may reduce oxidative stress within these cells, potentially improving mitochondrial MCSs. PPARγ exhibits anti-inflammatory properties and plays a key role in regulating lipid metabolism, which could offer therapeutic benefits in Charcot-Marie-Tooth disease, as neuroinflammation and oxidative stress are known contributors to peripheral nerve degeneration in this condition^58^. The peroxisomal rescue observed with Leriglitazone treatment and the morphological differences associated with the residual expression of *GDAP1* suggest that this protein may play a role in synthesizing peroxisomes that depend on mitochondrial membranes. Moreover, MFN2 and DRP1 interact with GDAP1^31,59^ and are MARCH5 interactors^60^. Thus, we speculate that GDAP1 could also be involved in the pathways of peroxisome biogenesis. Additionally, Leriglitazone has demonstrated promising outcomes in other neurodegenerative diseases, including Friedreich's ataxia or Rett syndrome^44,61,62^. Therefore, this work would open a window to a therapeutic possibility in patients with CMT due to *GDAP1* mutations.

Finally, the murine sciatic nerve study revealed that GDAP1 deficiency causes structural defects and lipid oxidation of the mitochondrial membrane. At the structural level, the GDAP1 nerve exhibited an abnormal morphology, increased variability in directionality, and a reduced number of nuclei, suggesting a disruption in communication between axons and Schwann cells. Additionally, the nodes of Ranvier displayed a greater length and abnormal morphology, which directly impacts nerve conduction^47^. These defects may contribute to denervation, leading to axonal atrophy at the neuromuscular junctions. Likewise, the distribution of mitochondria, lysosomes, and peroxisomes was abnormal in the absence of GDAP1. GDAP1 interacts with proteins involved in organelle transport, such as Tubulin, RAB6B, and Caytaxin^11^ and plays a role in mitochondrial movement along the axon and their proper positioning in the synaptic terminals^10^. In this study, we discovered that GDAP1 interacts with PEX14, a protein required for microtubule-based peroxisome motility^63^, suggesting a joint role of these proteins in peroxisomal movement. Further, the absence of GDAP1 causes redox impairment that correlates with phospholipid membrane oxidation in sciatic nerves. Altogether, these findings indicate that an imbalance in redox status within the axon, and likely at the mitochondrial MCSs, is a key factor contributing to the dysfunction associated with GDAP1 deficiency in CMT.

Summarizing, our findings suggest that GDAP1, a protein located at the outer mitochondrial membrane, facilitates the delivery and transport of organelles by mediating interactions between mitochondria, lysosomes, peroxisomes, and the cytoskeleton while maintaining the appropriate redox balance. Our findings highlight the critical role of GDAP1 in preserving MCSs and the structural integrity and functional capacity of peripheral nerves and propose PPARγ as a potential drug target for *GDAP1*-related Charcot-Marie-Tooth disease.

## Methods

### Ethical considerations and study approval

All procedures complied with the ethical guidelines of Sant Joan de Déu Children's Hospital and were approved by the Clinical Research Ethics Committee under reference PIC-223–19. Informed consent was obtained from the patients, patients' parents, or legal guardians. Experimental procedures followed the European Union Council guidelines (2010/63/EU) and Spanish regulations (RD 1201/2005). Research protocols with experimental mouse models are reported in detail to the local Ethical Committee of Animal Experimentation (CEEA) of the University of Barcelona before experiments start, and the Government of Catalonia has provided further approval. The registration number is C-GEN-354/22 for the *Gdap1*^−/−^ mouse model. All efforts were made to minimize pain and distress.

### Cell culture and transfection

#### Human fibroblasts:

age-matching control fibroblasts, GDAP1^Trp67Leu/Trp67Leu^ and MFN2^Arg104Trp/+^ fibroblasts were obtained from Sant Joan de Déu Children's Hospital Biobank. GDAP1^Gln163X/Thr288NfsX3^ [00CMT/000084F01] was obtained from CIBERER Biobank. Fibroblasts were cultured in Dulbecco's Modified Eagles's Medium high-glucose (Sigma-Aldrich) supplemented with 10% fetal bovine serum (FBS; Sigma-Aldrich), 2 mM L-glutamine (Sigma-Aldrich) and 100 mg/ml penicillin-streptomycin (Sigma-Aldrich) at 37°C in a 5% CO_2_ incubator. Cells were periodically tested for mycoplasma infection. According to the manufacturer's instructions, fibroblasts were transfected with Lipofectamine^™^ 3000 (Thermo Fisher Scientific, L3000015). The following plasmids were used: pCMV6-AC-Ø and pCMV-GDAP1-Myc plasmid^11^. Leriglitazone (Fisher Scientific) was resuspended in DMSO to obtain a stock solution at 10 mM. For leriglitazone treatment, fibroblasts were incubated for 48 hours with 500 nM leriglitazone in medium complemented with 1% DMSO.

#### Cell lines:

The SH-SY5Y neuroblastoma cell line was cultured in Dulbecco's Modified Eagles' medium/Nutrient mixture F-12 (Sigma-Aldrich) supplemented with 10% FBS, 2 mM L-glutamine, and 100 mg/ml penicillin-streptomycin at 37°C in a 5% CO_2_ incubator. According to the manufacturer's instructions, cells were transfected with FuGENE Transfection Reagent (Promega, E2312).

### Animal model and embryonic motor neuron (eMN) primary culture

*Gdap1* knockout (*Gdap1*^−/−^) mice were previously generated and characterized in our laboratory^48^. All the animals were kept under controlled temperature (23°C) and humidity (60%) on a 12-hour light/dark cycle with access to food and water ad libitum. eMN culture was prepared from 13.5 embryonic day (E13.5) mouse spinal cord as previously described^9,10^. Briefly, mouse embryo spinal cords were dissected, and the dorsal half was removed. Ventral spinal cords were dissociated mechanically after trypsin treatment (0.025% trypsin in HBSS) and collected under a 4% bovine serum albumin (BSA) cushion. The largest cells were isolated by centrifugation (10 minutes at 520 g) using iodixanol density gradient purification. The collected cells were finally suspended in a tube containing Neurobasal (Life Technologies) supplemented with B27 (Life Technologies), 2% horse serum (Life Technologies), 1x glutamate (Life Technologies), and a cocktail of recombinant neurotrophins: 1 ng/mL BDNF, 10 ng/mL GDNF, 10 ng/mL CNTF, and 10 ng/mL HGF (PreProtech). Isolated eMNs were plated on poly-DL-ornithine/laminin-coated surfaces and grown in a 5% CO_2_ incubator at 37°C. Media was changed every 2–3 days, and 2 μM AraC (Sigma-Aldrich) was added to the culture medium to limit the growth of non-neuronal cells.

### Western blot and co-immunoprecipitation assays (co-IP)

Fibroblasts were homogenized in lysis buffer (50 mM Tris HCl pH 7.4, 1.5 mM MgCl_2_, 5 mM EDTA, 1% Triton X-100, 50 mM NaF, and 1 mM Na_2_VO_3_) containing a protease inhibitor cocktail (Complete Mini-Protease Inhibitor Cocktail, Roche). Homogenates were centrifuged at 13200 rpm (FA-45–30-11 Rotor) for 15 minutes at 4°C, and the protein concentration of the supernatant was quantified by the BCA method (Thermo Fisher Scientific), resolved in sodium dodecyl sulfate-polyacrylamide gels (SDS-Page), and transferred onto PVDF Immobilon-P membranes (Merck). Membranes were blocked with 5% defatted milk or bovine serum albumin in TBS-0.1% Tween 20 buffer (25 mM Tris, 50 mM NaCl, 2.5 mM KCl, 0.1% Tween-20). Afterward, the membranes were blotted with the specific primary antibodies detected using secondary antibodies coupled to horseradish peroxidase. Proteins were processed for chemiluminescence with Amersham ECL Prime Western Blotting Detection Reagent (Cytiva) and visualized by iBright^™^ CL1000 Imaging System (Thermo Fisher Scientific). Band intensity was measured using ImageJ (NIH, http://rsb.info.nih.gov/ij).

For co-immunoprecipitation assays, 1 mg of total protein lysate was incubated with the specific antibody for 6 to 8 hours at 4°C, followed by overnight incubation with Protein G Sepharose^™^ 4 Fast Flow (Cytiva, GE17–0618-01) at 4°C. Beads were softly washed with lysis buffer, resuspended in Laemmli Buffer, heated at 95°C, and analyzed by SDS-Page and western blot.

### Immunofluorescence

#### Fibroblasts and cell lines:

Cells were seeded onto glass coverslips, washed with PBS, and fixed in pre-warmed 4% paraformaldehyde for 20 minutes at room temperature. Cells were permeabilized with 0.2% Triton in PBS for 30 minutes and blocked with 1% BSA and 4% serum in PBS. The specific primary antibodies were incubated overnight at 4°C, and the secondary conjugated antibodies were incubated for 1.5 hours at room temperature. The coverslips were mounted with Fluoromount-G with DAPI. To visualize mitochondria, cells were loaded with 200 nM MitoTracker Deep Red (Invitrogen, M22426) for 30 minutes at 37°C.

#### Embryonic motor neurons:

eMNs were washed with PB 0.1M and fixed in pre-warmed PHEM (PIPES 60 mM, HEPES 25 mM, EGTA 5 mM, MgCl_2_ 1 mM, 0.25% glutaraldehyde, 4% paraformaldehyde, and 4% sucrose) for 20 minutes at room temperature. Cells were permeabilized and blocked with BSA 4%—Triton 0.5% in PB 0.1 M for 1.5 hours. The following steps are explained above.

#### Sciatic nerve sections:

mice were sacrificed by cervical dislocation, and sciatic nerves were dissected and immediately frozen in liquid nitrogen and stored at −80°C. They were frozen in an OCT embedding medium and cut into 7 *μ*m sections on a cryostat. Nerve sections were fixed in PFA 4% for 7 minutes, washed with PBS-0.5% Tween-20, and blocked with 8% BSA and 1% Triton X-100 for 90 minutes at room temperature. Then, sections were washed with PBS-0.5% Tween-0.1% Triton and incubated with primary antibodies (in PBS, 5% BSA, and 0.1% triton X-100) overnight at 4°C. The next day, the slices were washed with PBS-0.5% Tween-0.1% Triton and incubated for 3 hours with secondary antibodies at room temperature. Finally, after several washes with PBS-0.5% Tween-0.1% Triton, the slices were mounted with Fluoromount-G with DAPI. For mitochondrial staining with anti-Cytochrome c antibody, PFA fixed tissues were permeabilized with 0.1% Saponin in PBS for 2 hours at room temperature. To block non-specific binding, nerves were incubated in 5% BSA containing 0.05% Saponin for 2 hours at room temperature. Tissues were then incubated with the antibody (in 5% BSA 0.05% Saponin) overnight at 4 °C. After several washes with PBS, the secondary antibody was incubated for an hour at room temperature. Finally, after several washes with PBS, the slices were mounted with Fluoromount-G with DAPI.

### Whole mount staining

This protocol was made as described by Dun et al.^45^. Mice were sacrificed by cervical dislocation, and sciatic nerves were dissected and washed in PBS. Nerves were fixed in PHEM (PIPES 60 mM, HEPES 25 mM, EGTA 5 mM, MgCl_2_ 1 mM, 0.25% glutaraldehyde, 4% paraformaldehyde, and 4% sucrose) for 5 hours at 4°C. Then, nerves were washed in PTX (1% Triton X-100 in PBS) three times for 10 minutes and incubated with blocking solution (10% FBS in PTX) overnight at 4°C. The following day, nerves were incubated with primary antibodies in a blocking solution for 72 hours at 4°C with gentle rocking. Then, nerves were washed with PTX three times for 15 minutes, then washed in PTX for 6 hours at room temperature (changing PTX every hour). Secondary antibodies and Hoechst dye (1:1000) were diluted in a blocking solution and incubated for 48 hours at 4°C. Next, nerves were washed in PTX three times for 15 minutes, followed by washing in PTX for 6 hours at room temperature (changing PTX every hour), and then washed overnight with PTX at 4°C. Finally, nerves were washed 3 times for 10 minutes in PBS. Nerves were cleared sequentially with 25%, 50%, and 75% glycerol in PBS between 12–24 hours for each concentration. Following clearing, nerves were mounted in CitiFluor (Agar Biosciences, 17970–25) for confocal imaging.

### Proximity Ligation Assay (PLA)

Fibroblasts were seeded onto glass coverslips, washed with PBS, fixed in pre-warmed 4% paraformaldehyde for 20 minutes, and permeabilized with ice-cold methanol at −20°C for 20 minutes. In eMNs, the permeabilization was performed with PBS 0.4% Triton X-100 for 10 minutes. After 1 hour of incubation at 37°C with the blocking solution in a pre-heated humidity chamber, cells were incubated overnight at 4°C with the specific primary antibodies. Afterward, we performed the PLA assay according to the manufacturer's instructions (Duolink *In Situ*-Fluorescence, Sigma-Aldrich), and the coverslips were mounted with Duolink *In Situ* Mounting Medium with DAPI.

### Antibodies

The following antibodies were used: ABCD3 mouse monoclonal (Santa Cruz, sc-514728), ACOX1 rabbit polyclonal (Proteintech, 10957–1-AP), β-ACTIN mouse monoclonal (Sigma-Aldrich, A5316), Caspr/paranodin/neurexin IV mouse monoclonal (Neuromab, UC Davis, Clone K65/35), Catalase mouse monoclonal (Santa Cruz, sc-271803), Catalase rabbit polyclonal (Thermo Fisher, 10HCLC), Cytochrome c rabbit monoclonal (Abcam, ab133504 ), GDAP1 rabbit polyclonal (Sigma-Aldrich, HPA024334 and HPA014266), GDAP1 mouse polyclonal (Abcam, ab194493), LAMP-1 rabbit polyclonal (Abcam, ab24170), MFN2 mouse monoclonal (Abcam, ab56889), Myc mouse monoclonal (Sigma-Aldrich, C3956), Nav1.6 (SNC8A) rabbit polyclonal (Alomone, ASC-009), PEX14 rabbit polyclonal (Proteintech, 10594–1-AP), PECI/EIC2 rabbit polyclonal (Proteintech, 20383–1-AP), S100β mouse monoclonal (Sigma-Aldrich, AMAb91038), TOMM20 mouse monoclonal (Sigma-Aldrich, WH0009804M1; BD Biosciences, 612278), and B3TUBB rabbit polyclonal (Sigma-Aldrich, T2200).

### Lipidomic studies

The lipidomics service of the Research Unit on Bioactive Molecules (RUBAM) at the Institute of Advanced Chemistry of Catalonia (IQAC/CSIC) performed the quantitative lipid analysis. Spinal cord and sciatic nerve samples from wild-type and *Gdap1*^−/−^ mice were dissected and processed with a potter in 20 mM Tris pH 7.8, centrifuged at 500g for 3 minutes, and stored at −80°C. Lipids were analyzed by performing liquid chromatography coupled with a high-resolution mass spectrometer (LC-HRMS) using an Acquity Ultra High-performance Liquid Chromatography (UHPLC) System connected to a TOF (Time of Flight) LCT Premier XE detector.

### RNA-seq and Bioinformatic Analysis

RNA was quantified by Qubit (Invitrogen), and quality was assessed with a Fragment Analyzer (Agilent), with the highest quality (typically RINs > 6, DV200 > 80%) utilized for library preparations. Fibroblast RNA was prepared and sequenced at the Mellowes Center for Genomic Sciences and Precision Medicine (RRID:SCR_022926) using the Illumina TruSeq RNA v2 library preparation kit and the Novaseq 6000 Sequencer with 101 bp paired end reads. Reads were aligned to the human reference transcriptome Gencode vM23 (GRCm38.p6) with at least 24 million mapped read pairs acquired per sample. Sequencing reads were processed through the Mellowes Center workflow, including MapRseq3^64^ and differential expression calculated by EdgeR^65^. Differentially expressed genes (DEGs) were filtered based on a false discovery rate (FDR) < 0.05 and an absolute FC ≥ |2.0| called between at least one condition and the reference control sample. Pathway analysis of DEGs was completed with RITAN (R package-rapid integration of annotation, network, and molecular database)^66^ and ShinyGO^67^, which query different annotation resources to analyze enrichment for an input set of genes. The Molecular Signatures Database (MSigDB) hallmark gene set collection^68^ was used as the annotation resource. Pathways with *q*-values < 2.5^E−3^ were considered enriched.

### Confocal and super-resolution imaging

Confocal and super-resolution microscopy analysis was performed by Leica TCS SP8 equipped with a white light laser and Hybrid spectral detectors (Leica Microsystems GmbH, Mannheim, Germany). The confocal images were acquired using an HC x PL APO 63x/1.4 oil immersion objective. Nodes of Ranvier were acquired using an HC x PL APO 100x/1.4 oil immersion objective and the HyD detector. DAPI was excited with a blue diode laser (405 nm) and detected in the 425–480 nm. Antibodies bonded to Alexa Fluor 488 were excited with an argon laser (488 nm) and detected in the 500–550 nm range. Antibodies bonded to Alexa Fluor 594 were excited with a white light laser (594 nm) and detected in the 610–795 nm. Z stacks of eight sections were acquired every 0.8 *μ*m along with the sample thickness. Appropriate negative controls adjusted confocal settings to avoid non-specific fluorescence artifacts. To compare the confocal data, identical confocal settings were used for image acquisition of different experiments.

#### Peroxisomal morphology analysis and soma-neurites segmentation.

The confocal pinhole was set to 1.0 Airy units. The original data was stored as 16-bit greyscale images with a spatial resolution of 1024×1024 pixels. Pixel sizes were 0.07×0.07 μm. Z-stacks were acquired in 0.8 μm z-increments along with the cell thickness. Maximum-intensity projections of image stacks of peroxisomes (red channel – PEX14), βIII tubulin (green channel), and nuclei (blue channel-DAPI) were calculated. The number of peroxisomes per cell and their roundness were quantified using ImageJ/Fiji (NIH). Oblate-prolate parameters were performed using the Imaris software (Bitplane, Switzerland). Stacks were reconstructed and visualized as three-dimensional (3D) volumes. Measurements of oblate and prolate were exported as morphological features. Prolate ellipticity is given a value from 0.01 to 1.00, with 1.00 representing an ellipsoid with one axis significantly longer than the others. Values closer to 0 represent a more spherical shape, while values closer to 1 represent a more elongated shape. Oblate ellipticity follows a similar behavior with the difference that 1.00 represents an ellipsoid with two axes equal in length but longer than the third, giving a more flattened shape. A custom 2-stage unsupervised segmentation method was implemented to discriminate between cell body and neurite regions. The first stage involved cell segmentation. First, image contrast was enhanced by linear gray-level transformation of the red channel (PEX14). Contrast limits for the input image were calculated by saturating the bottom 0.7 quantiles of all pixel values. Binarization obtained the autonomous delimitation of cell contour. Artifacts (objects<3.5 μm^2^) were then removed by a morphological area opening operation. Non-homogeneous staining can lead to the appearance of artifactual cell body holes. Morphology operations removed these: first, the total hole segments were obtained by subtracting the original binary image from the hole-filled (filling) image. Subsequently, a hole-free image was obtained by adding those segments smaller than a specified size (opening, ~ 15 μm^2^) to the original binary image. The second stage aimed to distinguish between cell soma and neurites properly. For that purpose, an Euclidean distance-based method capable of identifying structure thickness was used^69^. The approach started by identifying edges and contour standard directions using a Sobel operator (3×3). Next, a line was traced from each pixel, following the estimated direction to find all the intersection points. Euclidean distances with the intersection points were then calculated, keeping only the distance to the nearest intersected pixel. Cell bodies were subsequently defined by thresholding the distance map with a minimum thickness (Euclidean distance=100 pixels; 7 μm). The following steps involved structure dilation with a disk-shaped structuring element of radius 5 and an area filtering operation for removing small objects (200px, 3.5 μm^2^). Inward interpolation was then used to fill the regions where the initial foreground mask was specified—image binarization (Otsu's method) and, finally segmented cell somas. Neurites were identified from the difference between the initial binary mask and cell somas. For peroxisome recognition, the contrast of the red channel (PEX14) was enhanced by saturating the bottom 0.85 quantiles of all pixel values in the image. The 0.5 Gaussian (sigma5) filtered image was subtracted from the previously enhanced image to further decrease background illuminance. Thresholding by Otsu's segmentation followed by artifacts removal (objects smaller than 10 px/0.7 μm^2^) rendered the final binary mask, delimitating all the existing peroxisomes in the cell. Finally, the neurite delimiting mask obtained in the previous stages was applied to focus the subsequent peroxisome analyses exclusively in the peroxisomes localized in neurites (Supplementary Fig. 2). All the analyses were implemented and performed using the software MATLAB R2023b (The MathWorks Inc., Natick, MA, USA).

#### Mapping images.

For low-magnification extended-volume imaging (using the HC x PL APO CS2 10×/0.4 dry objective), we employed a high-precision motorized stage controlled by LASX Navigator software to capture large-scale 3D mosaics of each tissue section. The software automatically generated a list of 3D stage positions covering the volume of interest, calculated based on the dimensions of a single image in microns and the degree of overlap between adjacent images. Individual image tiles were sized at 800 × 800 pixels, with a z-step of 3.5–4 *μ*m (tissue thickness= 40–60 μm). Typically, 12–18 fields were captured for each extended image. Sequential excitation of sciatic nerves occurred at three different wavelengths: 405 nm, 488 nm, and 594 nm, corresponding to the excitation of DAPI, Nav1.6, and Caspr, respectively. DAPI emission was detected in the 425–480 nm range, Nav1.6 in the 500–550 nm range, and Caspr in the 605–795 nm range. Maximum projections were generated using LAS AF^™^ software (Leica Microsystems, Heidelberg, Germany). To ensure consistency and comparability of confocal data across different experiments, identical confocal settings were utilized for image acquisition in each figure.

#### Directionality and orientation analysis.

Directionality analysis was performed using Fiji and its Directionality plugin, which utilizes FFT and Gaussian fitting to determine peak positions. This algorithm selectively considers aligned elements in images, making it robust against random elements. The plugin aids in deducing structure orientation and generates histograms reflecting directional abundance. Isotropic images yield flat histograms, while those with preferred orientations exhibit defined peaks. The 'OrientationJ' plugin was also investigated, revealing that the structure tensor method yielded consistent dominant direction results akin to Fourier analysis using the 'Directionality' plugin.

#### Node of Ranvier analysis.

For the super-resolution images in nodes of Ranvier, Z stacks of 15 sections were acquired every 0.35 μm. Stacks were reconstructed and visualized as three-dimensional (3D) volumes with Imaris software (Bitplane). The confocal pinhole was set to 1.0 Airy units. The original data was stored as 8-bit greyscale images with a spatial resolution of 1024×1024 pixels. Pixel sizes were 0.05×0.05 μm. Z-stacks were acquired in 0.35 μm z-increments along with the structure thickness. Maximum-intensity projections of image stacks of the paranodes (red channel, Caspr), nuclei (blue channel, DAPI), and nodes (green channel, Nav1.6) were calculated. First, the image contrast of the green channel (nodes) was increased by saturating the bottom 10% of all pixel values. Otsu's binarization obtained the autonomous delimitation of the nodes. Morphological opening was the eligible technique for removing unwanted minor artifacts (100px, <5 μm^2^) from the binarization process. The binary mask delimiting the node of Ranvier already enabled the number of fragments conforming to the whole structure to be calculated. Other biomarkers were also calculated further to assess potential node differences between wild-type and knockout groups. First, the position and size of the smallest box containing the node (bounding box) were obtained. Thus, node symmetry was evaluated by the Sørensen–Dice coefficient of the original binary node and its specular image (flipped along the bounding-box major axis). The node/bounding-box area ratio was also analyzed to measure solidity (node compaction, density…) (Supplementary Fig. 4). The node length was measured with Leica LAS X software. All the analyses were implemented and performed using the software MATLAB R2023b (The MathWorks Inc., Natick, MA, USA).

### Intracellular pH measurements *in vivo*

For live-cell imaging, control fibroblasts were plated on 35-mm Ibidi dishes (Ibidi GmbH, Martinsried, Germany), and 24 hours later, cells were transfected with the plasmid GPI-RpHLuorin2 (#171721, Addgene), which contains the gene GPI for Glycosylphosphatidylinositol, a plasmatic membrane protein, fused with the pHluorin2 pH fluorescent indicator. After 48 hours, plates were washed with the more alkaline buffer and incubated for 2 minutes with the corresponding buffer. Fluorescent images were acquired using confocal microscopy with an HC PL APO CS2 100x/1.40 Oil objective. pHluorin2 was excited in two channels, one with a blue diode laser at 405 nm and the other with a white laser at 475 nm, and both were detected in the 500–560 nm range. Lysotracker Red was excited with a white light laser (594 nm) and detected in the 610–795 nm range.

#### pH Calibration curve buffers.

Given the experimental data, a pH calibration curve was generated following a previously described methodology^70^. The buffers for generating it contain 125 mM KCl, 25 mM NaCl, 10 μM monensin, and 25 mM N- [2-hydroxyethyl]-piperazine-N-[2-ethanesulfonic acid] (HEPES, pH 8.0, 7.5 or 7.0) or 25 mM 2-[N-morpholino] ethanesulfonic acid (MES, pH 6.5, 6.0, 5.5, 5.0, 4.5, 4.0, or 3.5). Each buffer solution is adjusted to the appropriate final pH using 1 N NaOH or 1 N HCl. Cells were sequentially incubated in a different buffer at pH 3.5, 4.0, 4.5, 5.0, 5.5, 6.0, 6.5, 7.0, 7.5, 8.0. Cells were incubated in buffers for 5 minutes before the measurement to obtain a calibration conversion formula for each imaging experiment (Supplementary Fig. 3).

#### pH measurement in mitochondria-lysosome MCSs.

To obtain the TOMM20-pHLuorin2, we clone the outer mitochondrial protein TOMM20 (mApple-TOMM20-N-10, # 54955, Addgene) into the plasmid pRpHluorin2-N1 (#171716, Addgene) using the restriction enzymes NheI and BamHI (Thermo Scientific). Fibroblasts were plated on 35-mm Ibidi dishes (Ibidi GmbH, Martinsried, Germany), and 24 hours later, cells were transfected with the plasmid TOMM20-pHluorin2. After 48 hours, cells were incubated with 75 nM LysoTracker Red DND-99 for 30 minutes at 37°C, washed, and incubated with DMEM without phenol red to obtain the images.

#### Data analysis/pHluorin2 image analysis.

The cells' images under specific pH conditions were analyzed with Image-J software (National Institutes of Health, Bethesda, MD, USA) to compute the pH calibration curve. Two images were acquired for each sample at long (475 nm) and short (405 nm) wavelengths. From them, the mean intensity of the cell membrane (ROI: 2×0.5 μm) with the corresponding background subtraction when using both the long and short wavelengths was computed to perform a ratio between them:

$$\text{Ratio}=\frac{I_{-}m_{\text{long}}\;-\;I_{\_}b_{\text{long}}}{I_{-}m_{\text{short}}\;-\;I_{\_}b_{\text{short}}}$$

With I_m being the mean fluorescence intensity and I_b being the background mean intensity. The subindex long and short refer to the pHluorin2 wavelengths excitation. This ratio was used with the known pH values to establish the calibration curve. An iterative z-score-based statistic study was computed to remove outliers and achieve stabilization. A satisfactory 4th-grade polynomic calibration curve was generated and later used to determine mitochondrial membrane pHs from confocal acquisitions. The pHluorin2 fluorescence ratio for each mitochondria-lysosome contact object was calculated similarly. pH values for each contact object were then calculated, assuming a relationship between pH and pHluorin2 fluorescence ratio.

### Statistics

Data are expressed as mean ± standard deviation (SD) or box plots showing the median, box edges represent the [25^th^ and 75^th^ percentiles], and the whiskers extended to the minimum and maximum values. Individual values are displayed as dots. The Kolmogorov-Smirnov test assessed the normality of data. Statistical analysis was performed using GraphPad Prism (version 10; GraphPad Software, Inc., La Jolla, CA, USA) with a minimum of three independent experiments. The specific test applied in each case is indicated in the figure legend. P-values less than 0.05 were considered significant. P-values are indicated by asterisks *P < 0.05, **P < 0.01, ***P < 0.001.

## Supplementary Material

Supplement 1
