# Supplementary material for: Abnormal redox balance at membrane contact sites causes axonopathy in GDAP1-related Charcot-Marie-Tooth disease
Source: Res Sq. 2024 Dec 31:rs.3.rs-5682984. Preprint. [Version 1] doi: 10.21203/rs.3.rs-5682984/v1 (PMC11722552; doi:10.21203/rs.3.rs-5682984/v1)
Supplement: Supplement 1 [file NIHPPRS5682984V1-supplement-1.pdf]

a

	67	163	288
H. sapiens	...SEHNEPWFMRLN...	...TRIRSQIGNTESELK...	...ERVLKRKTFNKVLGHVNN...
M. musculus	...SEHNEPWFMRLN...	...TRIRSQIGNTESELK...	...ERVLKRKTFNKVLGHVN...
R. norvegicus	...SEHNEPWFMRLN...	...TRIRGQIGNTESELK...	...ERVLKRKTFNKVLGHVN...
B. taurus	...SEHNEPWFMRLN...	...TRIRSQIGNTESELK...	...ERVLKRKTFNKVLGHVNN...
D. rerio	...SEHNEPWFMRLN...	...THIRTQIGNTESELK...	...ERVLDRPTFRRVLGHVNN...

b

Gene (Ref. Sequence)	Nucleotide (amino acid) change	gnomAD/CSVS frequency	Variants classification (ACMG)	PREDICTORS OF PATHOGENICITY			
				PROVEAN (score)	Mutation taster (P-value)	DANN (score)	CADD (score)
GDAP1 (NM_018972.4)	c.200G>T (p.W67L)	NR/NR	Uncertain significance	Damaging (-10.85)	Disease causing (1)	Damaging (0.99)	34
	c.487C>T (p.Q163X) / c.863insA (p.T288NfsX3)	0.000015/NR / NR/NR	Pathogenic / Pathogenic	Neutral (-2.42) / -	Disease causing (1) / -	Damaging (0.99) / -	42 / -

c

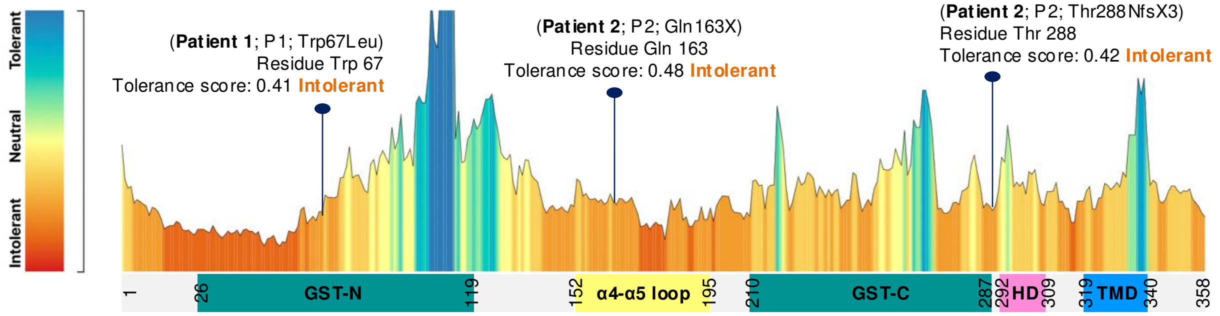

d

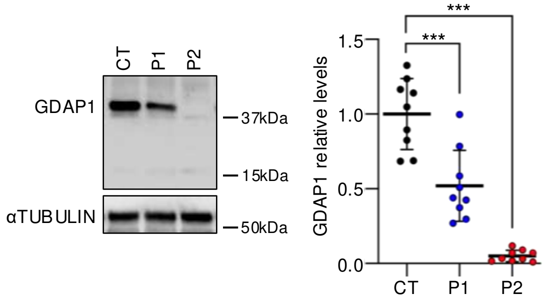

Supplementary Fig. 1. *In-silico* biology study of *GDAP1* clinical variants.

a, Multiple sequence alignment of GDAP1 showing the amino acid positions 67, 163 and 288 in different vertebrate species. **b,** Predicted effect of *GDAP1* clinical variants. Mutation taster and DANN (with scores ranging from 0 to 1, where 1 is predicted the most damaging), SIFT (with scores ranging from 1 to 0, where 0 is predicted the most damaging), PROVEAN (with scores equal or below -2.5 being deleterious and above -2.5 being neutral), CADD (Combined Annotation Dependent Depletion) (with scores ≥ 20 indicating that the variant is predicted to be among the 1% most deleterious substitutions in human genome) and missense variation score (positive scores indicate intolerance to variation and negative scores are given to genes that had more variants than expected) values of gnomAD database. Allele frequency in total population (gnomAD; Genome Aggregation Database) and in Spanish population (Collaborative Spanish Variant Server; CSVS). Genetic variants have been classified following the American Collage of Medical Genetics and Genomics (ACMG) guidelines, using VarSome (last accessed July 2024). **c,** An intolerance landscape plot generated by MetaDome for *GDAP1* clinical variants (top panel) and a schematic outline of GDAP1 protein showing its domains (Uniprot: Q8TB36) (lower panel). The location of patient 1 (P1) and patient 2 (P2) clinical variants is shown. GST-N, Glutathione-S transferase N-terminal; GST-C, Glutathione-S transferase C-terminal; HD, hydrophobic domain; TMD, transmembrane domain. **d,** GDAP1 protein levels in control, P1 and P2 fibroblasts by western blot. Quantification is shown in the right panel. Data represents mean ± SD and individual values are displayed as dots (n=9). One-Sample t test. ***$p<0.001$.

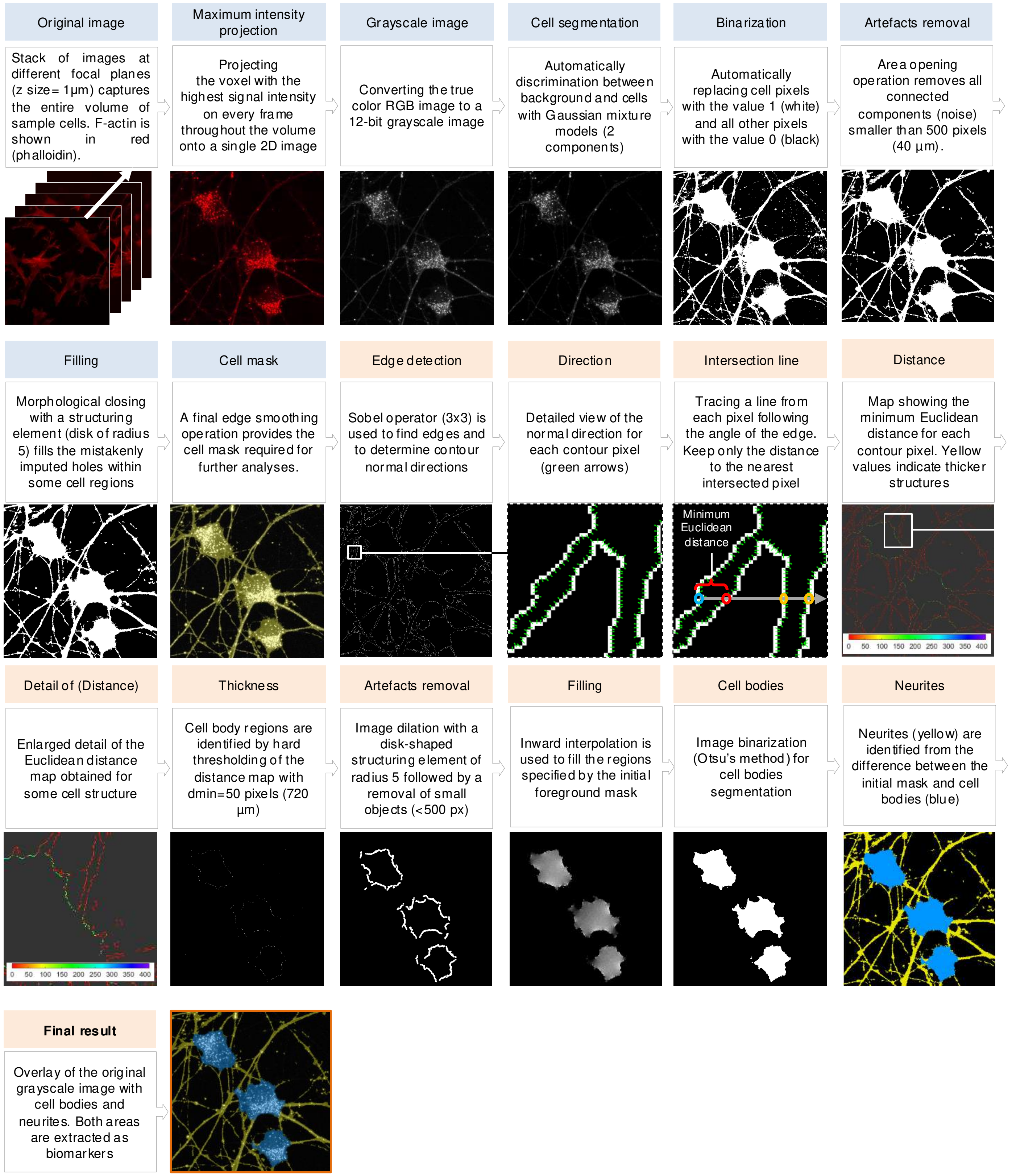

Supplementary Fig. 2. Overview of the automated cell segmentation workflow the discrimination between neurites and soma regions. Image segmentation creates a pixel-wise mask delimiting cell region (blue titles). Subsequently, the algorithm automatically identifies soma and neurites regions on a cell (orange titles), based on the structure thickness. The ratio neurites (yellow)/somas (blue) area is then computed and used as a marker of cell differentiation for group comparisons.

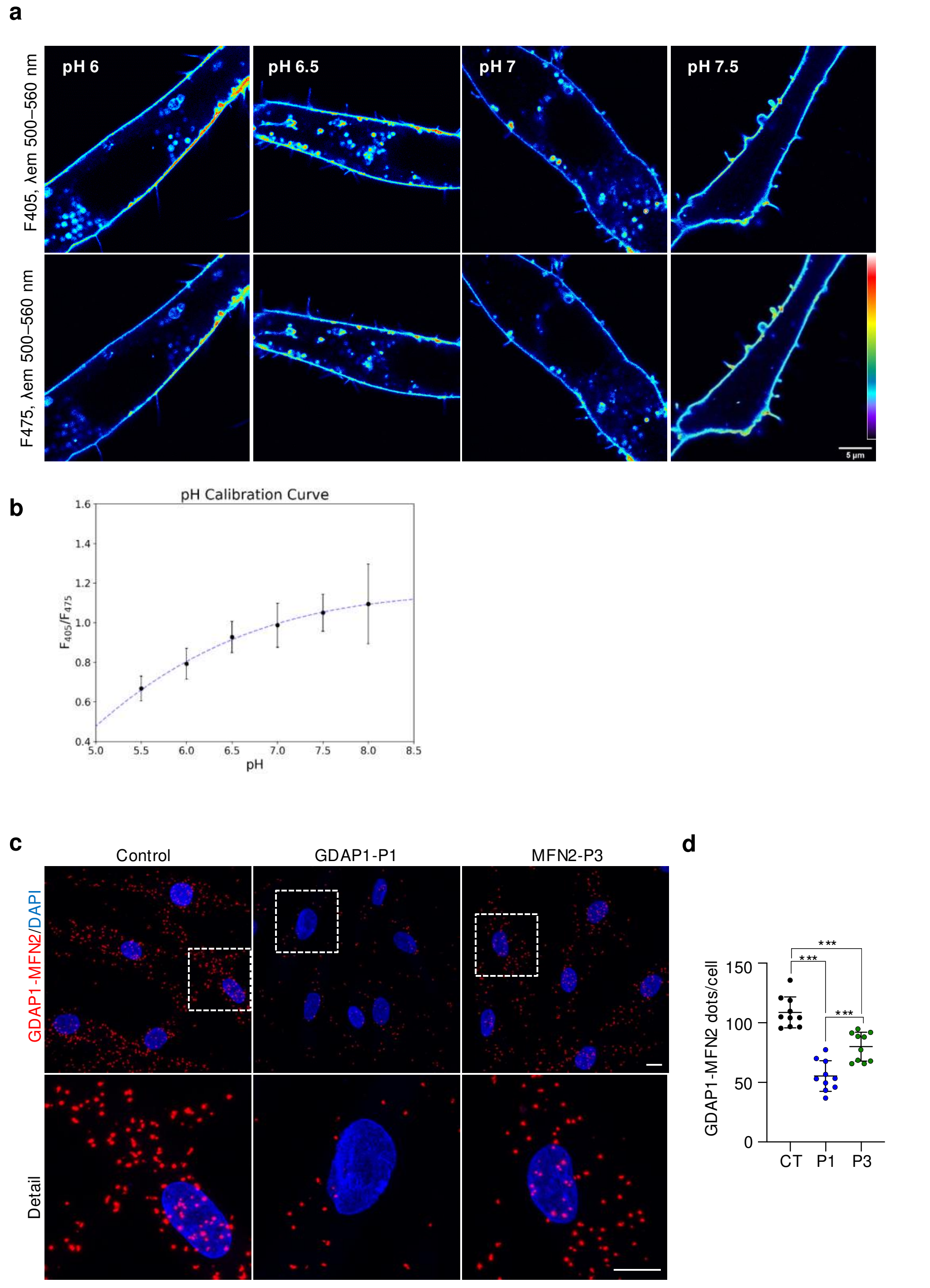

Supplementary Fig. 3. ***In Situ* pH calibration of pHluorin2 in live fibroblast membranes. a,** Representative confocal images of fibroblasts expressing GPI-RpHLuorin2 (plasmatic membrane) at different pH levels are shown in defined calibration buffers (n = 3, 389 events in total). Sequential dual-excitation confocal imaging was used for the measurements. The pseudocolor scale is shown at the bottom right. Warm colors such as white and red represent maximum intensities, whereas cold colors like blue are representative of low intensities. Scale bar = 5 *µ*m. **b,** The pH titration curve of the pHluorin2 obtained from the fluorescence emission intensity ratio F_{exc405}/F_{exc475} as a function of pH is presented. The fluorescence ratio was plotted between pH 6.0 and 7.5, and the ratio for each pH condition in the membrane was calculated and fitted to the curve. The graphical nonlinear fit (sigmoidal dose-response) was performed using GraphPad Prism software. The error bars represents the standard deviation of the measurements. [$y=0.0069x^3-0.1927x^2+1.817x-4.655$]. **c,** PLA assay between endogenous GDAP1 and MFN2 in control, GDAP1-P1 and MFN2-P3 patients' fibroblasts. A detail is shown. Scale bar: 10 µm. **d**, Quantification of the number of GDAP1-MFN2 dots per cell. One-way ANOVA followed by Tukey's post hoc (CT=64, P1=58, P3=50,fibroblasts, three independent experiments).

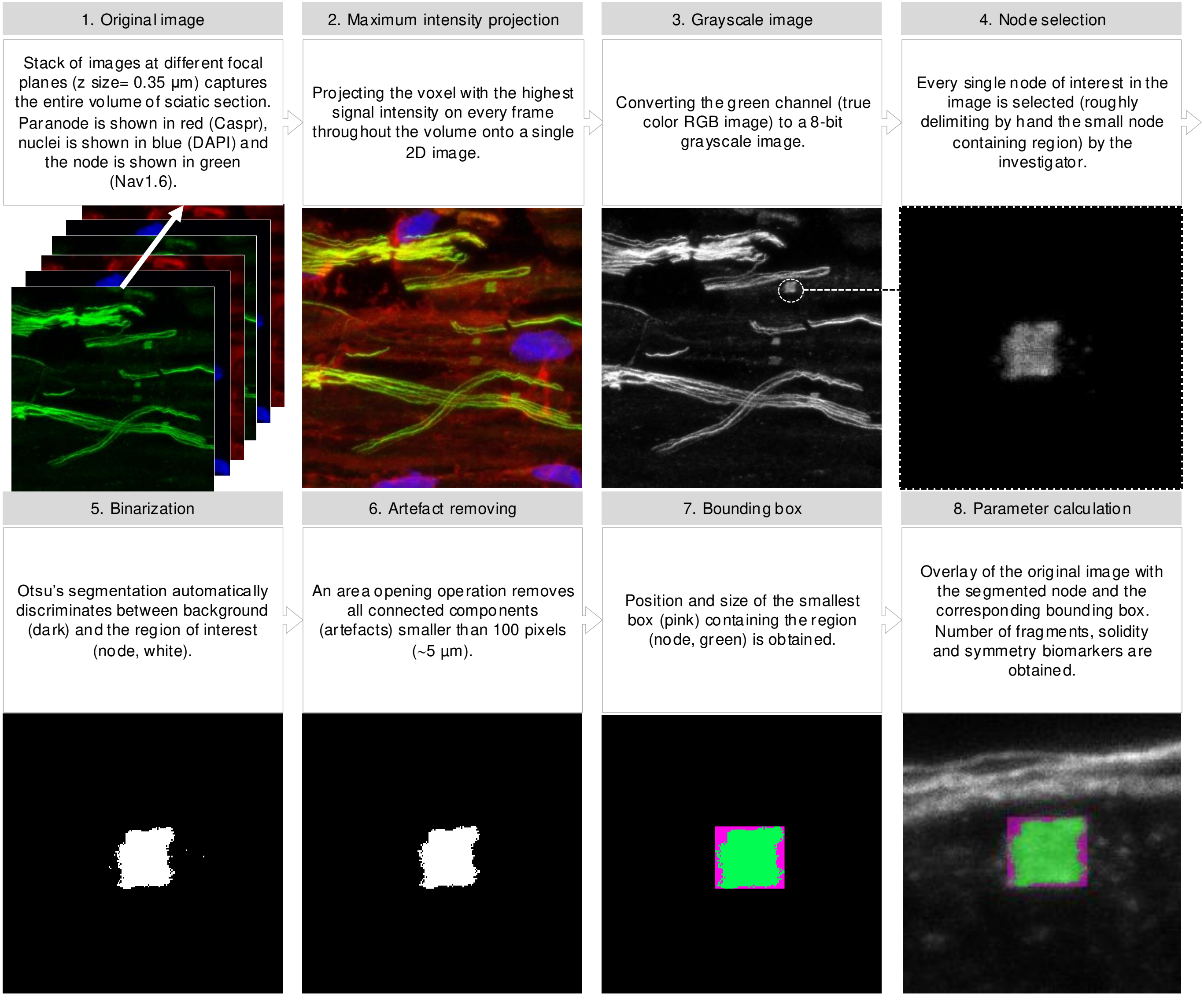

Supplementary Fig. 4. Detailed steps for the automatic identification and assessment of the Caspr-labelled paranodes in the sciatic nerves. A two-stage semi-automatic algorithm is able to identify and assess paranodes: the first stage comprises a series of steps involving image segmentation, which discriminates the region of interest (paranode) from the background. The second stage identifies the "bounding box", that is, the smallest rectangle (pink) enclosing all boundaries indexed by the elements of the region of interest (paranode, green). This measurement allows the calculation of several parameters such as, solidity and symmetry, for the appropriate comparisons between cell lines.
